# Dietary Carbohydrate Intake and Skeletal Muscle Glycogen: A Systematic Review and Meta‐Analysis to Determine Optimal Carbohydrate Loading Strategies

**DOI:** 10.1111/sms.70379

**Published:** 2026-10-03

**Authors:** R. O. Jones, J. L. Areta, H. O. Fortis, J. Pugh, J. B. Louis

**Affiliations:** ^1^ Research Institute for Sports and Exercise Sciences (RISES), Liverpool John Moores University Liverpool UK; ^2^ College of Medicine and Public Health Flinders University Bedford Park South Australia Australia

## Abstract

A high dietary carbohydrate intake in the days pre‐competition is recommended to optimize glycogen stores for endurance exercise performance. However, previous reports show high variability in muscle glycogen concentrations between individuals following similar CHO intakes. Therefore, the amount of CHO, duration, and amount of exercise required to maximize glycogen stores pre‐competition remains unclear. The aim of this systematic review and meta‐analysis was to determine the relationship between dietary CHO availability and skeletal muscle glycogen, whilst identifying the effect of other covariates on muscle glycogen concentration. The analysis included trials (randomized or non‐randomized from five online databases) published until June 2026, that reported CHO intake and exercise for ≥ 24 h prior to collection of muscle samples using biopsies to measure skeletal muscle glycogen. Included studies were assessed descriptively and quantitatively using linear and non‐linear regression, and generic inverse‐variance random‐effects meta‐analyses, including subgroups to investigate heterogeneity. Descriptive synthesis included 63 trials (*n* = 641 participants), 18 were included within the meta‐analysis. Exploratory linear regression indicated a significant relationship between relative CHO intake and whole muscle glycogen for recreationally active (*r* ^2^ = 0.332, *p* < 0.001) and endurance trained individuals (*r* ^2^ = 0.490, *p* < 0.001). Meta‐analyses revealed increased dietary CHO intake from a low‐moderate (< 6.5 g·kg^−1^·day^−1^) to a high‐very high intake (> 6.5 g·kg^−1^·day^−1^) significantly increased muscle glycogen concentrations (+191.9 mmol·kg^−1^ DM, 95% CI from 142.8 to 241.1 mmol·kg^−1^ DM, *p* < 0.0001) but displayed significant heterogeneity (*p* < 0.0001, *I* ^2^ = 94%). The delta CHO intake between study conditions (expressed as relative g·kg^−1^·day^−1^ and absolute g·day^−1^) and duration of CHO loading interventions suggested a significant subgroup effect (*p* < 0.05) but failed to explain the heterogeneity in muscle glycogen concentration. Glycogen concentration primarily depends on quantity of CHO intake, with period of increased CHO intake, participant training status, and exercise all being key effectors. Based on current findings, to significantly increase pre‐competition muscle glycogen stores athletes should consume > 8 g·kg^−1^·day^−1^ for 36–48 h pre‐competition, with prior glycogen depleting exercise combined with consumption of high glycaemic index CHO allowing more rapid enhancement of stores (24–36 h). However, significant heterogeneity and confounding variables in previous research introduce uncertainty in these recommendations, and further well‐controlled studies are required to determine the true optimal pre‐exercise CHO intake to maximize glycogen concentrations.

## 1 Introduction

Endogenous stores of dietary carbohydrate (CHO), in the form of muscle glycogen, are the primary fuel source during moderate‐to‐high‐intensity endurance exercise [1, 2]. Limited stores within skeletal muscle and the liver (~400 and ~100–120 g, respectively, at rest under “normal” physiological conditions) are rapidly depleted during endurance sports competitions, leading to a reduction in physical work capacity due to reduced substrate availability for high‐intensity exercise [3, 4, 5]. As such, endurance athletes increase dietary CHO intake and decrease training load in the days pre‐competition to maximize glycogen storage within skeletal muscle, a strategy termed CHO loading [3, 6, 7].

CHO loading was first introduced by Scandinavian researchers in the 1960s [6], shortly followed by the introduction of the “classic” strategy, where two exhaustive exercise sessions separated by 72 h of low dietary CHO intake were followed by 72 h of rest and a high CHO intake (~575 g per day [g·day^−1^]). This resulted in above‐normal muscle glycogen stores (~800 mmol kg^−1^ of dry muscle mass [DM]) [3], a phenomenon now termed “muscle glycogen supercompensation.” CHO loading strategies have since been modified and updated, suggesting that shorter periods (24–48 h) of very high CHO intakes, without exhaustive exercise, can be equally effective in achieving enhancement of muscle glycogen storage [8, 9, 10]. As such contemporary nutritional guidelines recommend 10–12 g of CHO per kg of body mass per day (g·kg^−1^·day^−1^) for 36–48 h pre‐endurance competition, to maximize muscle glycogen concentrations and enhance subsequent endurance exercise performance [11].

Despite over half a century of research, and the importance of a high CHO intake for supercompensation of muscle glycogen stores being well‐established [3, 8, 9, 10, 11], until recently, a systematic review and meta‐analysis that objectively quantified the effectiveness of nutritional interventions to maximize muscle glycogen stores had never been conducted [12]. This is particularly relevant, because even though CHO loading is a practice well‐established and commonly advocated [11], its effectiveness in maximizing muscle glycogen concentration is still not completely clear (particularly for CHO loading durations of 24–72 h, which was beyond the inclusion criteria of the previous review [12]). For example, studies that utilized similar CHO loading protocols (~8 g·kg^−1^·day^−1^ for 72 h) in endurance trained participants have reported high variability of muscle glycogen concentration, with values ranging from 450 to 800 mmol·kg^−1^ DM [3, 13, 14, 15]. The reasons for such variability are unclear, as well‐established effectors of glycogen synthesis (CHO quantity, loading duration and training status) were matched [16, 17, 18]. Therefore, a better understanding of the effectors which influence glycogen stores is required to determine optimal CHO loading strategies. As such, this systematic review and meta‐analysis aimed to examine the relationship and effect of dietary CHO intake on muscle glycogen, and to characterize covariates of muscle glycogen concentration.

## 2 Methods

This systematic review and meta‐analysis was registered on the Open Science Framework in December 2023 (https://osf.io/bdasy/). The Preferred Reporting Items for Systematic Reviews and Meta‐analysis Statement (PRISMA) and Cochrane handbook [19, 20] were consulted throughout the review protocol. Peer‐reviewed, English‐language journal articles from any time period were included, whilst conference proceedings were excluded due to insufficient information. The inclusion criteria were as follows:

- Population—Healthy human, male and/or female participants (aged 18–60 years).
- Intervention—Carbohydrate intake (relative to body mass [g·kg^−1^·day^−1^] or calculable as such) and exercise status reported for ≥ 24 h pre‐biopsy.
- Comparisons—Different carbohydrate intakes, loading durations (e.g., muscle biopsies following 24 vs. 72 h), participants or conditions.
- Outcome—Whole skeletal muscle glycogen reported in dry mass or calculable as such, analyzed from muscle samples obtained using muscle biopsies and biochemical analysis.
- Study design—Randomized, counterbalanced, or non‐randomized controlled trials.

Any studies that failed to meet the above criteria were excluded. As there is limited data to determine how age influences the ability to achieve glycogen supercompensation, the population age range was estimated. Children/youth participants were excluded due to hormonal and metabolic differences versus adults, which may influence glycogen storage. No exclusion criteria were specified for muscle biopsy location, yet only *vastus lateralis* and *gastrocnemius* biopsies surpassed the screening process. Non‐randomized trials were included to incorporate all relevant information within qualitative descriptive synthesis.

### 2.1 Search Strategy and Yield

Five online databases (PubMed, Web of Science, Cochrane, SportDiscus and Scopus) were independently searched by two researchers in February 2022 and December 2024 (R.O.J. and H.O.F., respectively), using the following combination of key words: (CHO OR carbohydrate OR maltodextrin OR glucose OR sucrose OR fructose) AND (Load* OR supercompensat*) AND (muscle OR glycogen OR stores OR storage) AND (concentration OR content OR synthesis OR resynthesis OR utilization OR accumulation). The search was updated again in June 2026, to ensure all relevant studies were captured. No database filters or limits were used to include all potential studies within the screening process. The keyword algorithm from each database for the latest search is reported within Supporting Information. Full search records were exported to Endnote citation manager (version 21, Philadelphia, USA) for title and abstract screening. Any discrepancies were discussed until agreement.

### 2.2 Data Extraction

Articles which qualified following title and abstract screening were read in full, with relevant study data extracted onto a custom Microsoft Excel spreadsheet (Microsoft, California, USA). In line with the PICO terms (Population, Intervention, Comparison, Outcome), data surrounding participants (sample size, sex, age, training level, V̇O_2max_), CHO loading interventions (glycogen depletion/exercise protocol, relative and absolute CHO intake, CHO type [food versus supplements and glycaemic index], CHO loading protocol duration, exercise conducted during loading days), study outcome (whole muscle glycogen across multiple timepoints) and study design were all extracted. Other potentially important variables, such as muscle biopsy method (Bergström, Weil‐Blakesley conchotome or gun) and location, biochemical analysis technique, and study dietary control method were also extracted. Participant training status/performance level was categorized as untrained, recreationally active, trained, well‐trained, and elite using criteria outlined by De Pauw et al. [21] and Decroix et al. [22] for males and females, respectively. Decisions were made based on available information reported within published articles. If limited information was available, provided ≥ 2 of the performance level criteria were met, participants were categorized accordingly. If no information was available to provide a judgment, author description was accepted. It was noted whether studies reported V̇O_2max_ or V̇O_2peak_, however, for training status classification, both were considered as V̇O_2max_ [23].

All muscle glycogen values were presented as means ± SD or SE, either in text, tables or figures, apart from Jensen et al. [24] where glycogen was reported as median and interquartile range. To quantify data points presented exclusively in figures (*n* = 17 studies [10, 13, 15, 16, 25, 26, 27, 28, 29, 30, 31, 32, 33, 34, 35, 36, 37]), an online plot digitizer was used (Plot Digitizer, POrbital, Mumbai, India), which was tested for accuracy using studies containing data presented in text and figures, which generated a mean difference of 2.2 ± 5.5 and 5.5 ± 7.5 mmol·kg^−1^ DM for studies originally presented as mmol·kg^−1^ DM and those converted from mmol·kg^−1^ wet mass to DM, respectively [9, 10, 15, 25, 38, 39, 40, 41, 42]. Data not presented as mmol·kg^−1^ DM were converted using previously established conversion factors [43].

### 2.3 Descriptive Methods

The literature search and descriptive synthesis aimed to collect robust evidence within the topic, whilst describing different participant and methodological characteristics between studies. As such, descriptive analysis used tallies of extracted data, which were conducted in two stages by an individual researcher. The first stage focused on study and participant characteristics whilst the second focused on differences between study exercise interventions, CHO loading protocols and muscle biopsy measures. Tallies were totaled and repeated by the same researcher to ensure accuracy, with any discrepancies repeated a third time. To compare differences in CHO intake a low, moderate, high and very high relative CHO intake was defined as < 3, 3–6.5, > 6.5–9 and > 9 g·kg^−1^·day^−1^ (adapted from Areta and Hopkins [43]).

### 2.4 Exploratory Linear Regression

To visually describe the relationship between dietary CHO intake (relative and absolute) and skeletal muscle glycogen, exploratory simple linear and nonlinear 2nd degree polynomial regressions were conducted using information included within the descriptive synthesis. Individual study groups/conditions were plotted based on CHO intake during the intervention and muscle glycogen, separated by participant training status (recreationally active, endurance trained and well‐trained). Study conditions that received additional interventions that significantly influenced muscle glycogen concentrations, such as multiple biopsies in close anatomical proximity [44], creatine ingestion [45], delayed CHO refeed [27] or repeated glycogen depletion [46] were excluded, whilst control/placebo conditions were included provided inclusion criteria were satisfied. Studies with multiple timepoints were included as separate groups, as such all observations were not statistically independent. Sensitivity analyses were conducted to determine effects of including all types of study design within the same regression model, the best fit nonlinear polynomial model and to confirm results were robust to the violated statistical assumption of independence of observations (see Supporting Information).

### 2.5 Meta‐Analysis Methods

To quantify meaningful differences between dietary CHO intake and skeletal muscle glycogen refined inclusion criteria were introduced to primarily include robust study protocols with low risk of bias (≤ 1 risk of bias domain judged as some concerns; see Section 2.6) within the meta‐analysis. As such, studies were required to be randomized controlled trials where a low‐moderate CHO intake control was compared against an intervention group which consumed a high‐very high relative CHO intake (0–6.5 vs. > 6.5 g·kg^−1^·day^−1^, respectively). CHO intake was required to be consistent throughout the loading period with total loading duration matched between conditions (e.g., 3 g·kg^−1^·day^−1^ for 48 h vs. 8 g·kg^−1^·day^−1^ for 48 h).

The meta‐analysis was conducted using a generic inverse‐variance random‐effects model on RevMan (version 5.4.1, Copenhagen, Denmark), where a mean difference and SE of the mean difference were calculated for each pairwise comparison. As magnitude of treatment effect can be influenced by experimental design, parallel group and crossover trials were analyzed separately [47]. For crossover trials (*n* = 12) dependency was accounted for using previously described methods [48]. Three studies reported individual participant data within original articles [49, 50, 51], remaining authors were contacted, and a further two provided individual participant data [14, 52]. For the remaining seven crossover trials, a pooled correlation coefficient was used based on the available calculated correlation data [14, 49, 50, 52]. Crossover trials with multiple eligible comparisons (i.e., four conditions which allowed two comparisons of a low‐moderate vs. high‐very high CHO intake) were included separately, and sensitivity analyses were conducted to confirm this did not impact results in practice (see Supporting Information, Table S3; [19]). As Jones et al. [51] contained three conditions (moderate, high and very high CHO), the high and very high CHO groups were combined for the overall effect estimate, to include a single pairwise comparison [19, 48]. Two mixed design trials were included within the quantitative synthesis, where two separate groups (males and females) were compared across repeated measures of manipulated CHO intake. Groups within the same defined category of CHO intake were combined using previously described methods (see Supporting Information; [19]) and pairwise comparisons were then treated as crossover [53] or parallel group trials where appropriate [54]. Sensitivity analyses were conducted to determine the effects of choosing a fixed versus random effects model and ensure borrowed correlation coefficients did not significantly impact results (Supporting Information; Table S4).

Authors pre‐identified the differences in CHO intake between conditions (expressed as relative and absolute intake), duration of manipulated CHO intake (loading duration), participant training status and sex, and muscle biopsy location as variables that could explain predicted study heterogeneity, which was explored with subgroup analyses exclusively in randomized crossover design studies. Pre‐analysis, subgroup comparisons between different defined levels of CHO intake (e.g., low versus high, moderate versus high, moderate versus very high) were planned, however this analysis was not deemed appropriate due to vastly uneven covariate distributions (2, 3, 5, and 11 comparisons included within each subgroup) and impaired sensitivity. Therefore, subgroups for the delta (Δ) in relative and absolute CHO intake between low‐moderate versus high‐very high CHO loading interventions were explored, with subgroups specified at 1 g·kg^−1^·day^−1^ increments from 1.5 g·kg^−1^·day^−1^ and 100 g increments from 100 g·day^−1^ of CHO, respectively. These increments were pre‐determined based on the possibility to result in a small but worthwhile change in glycogen concentration. An analysis for both relative and absolute CHO was conducted to explore whether differences occurred based on how CHO intake was expressed, and whether an absolute CHO threshold for glycogen supercompensation existed. For subgroups of ΔCHO intake, Jones et al. [51] was entered as multiple comparisons across separate subgroups (moderate vs. high and moderate vs. very high) to explore the differences between a moderate vs. high and moderate vs. very high CHO intake. The number of subgroups was determined post hoc as this was dependent on study interventions. Statistical significance was set at *p* < 0.10 for heterogeneity (i.e., variability in the intervention effect between studies) determined using the Cochran's *Q* test, to account for limited power when relatively few studies are included. The variability in the effect estimate caused by heterogeneity was described using the *I* ^2^ statistic, with scores of 0%–40%, 30%–60%, 50%–90%, and 75%–100% corresponding to potentially unimportant, moderate, substantial and considerable heterogeneity, respectively [19].

### 2.6 Risk of Bias Assessment

All trials were assessed for risk of bias using the Cochrane risk of bias tools for randomized crossover and parallel group trials (RoB 2 [55]), and non‐randomized trials of interventions (ROBINS‐I [56]). Assessments were conducted on a whole study level across six domains using both tools (RoB 2 included randomization, carryover effects, deviations from intended interventions, missing outcome data, measurement of the outcome and selection of reported results; ROBINS‐I included confounding, classification of interventions, participant selection, missing data, measurement outcome and selection of reported results). Each domain contained a series of signaling questions answered “yes,” “probably yes,” “no,” “probably no,” or “no information.” The risk of bias categorized for each domain (“low risk,” “some concern,” or “high risk” for RoB 2 and “low,” “moderate,” “serious,” and “critical” risk for ROBINS‐I) was judged by researchers and later confirmed by a pre‐determined algorithm. Overall, risk of bias was based on the highest risk score across all domains. Assessments were completed by two researchers independently (R.O.J. and J.B.L.) with results compared, and any disagreements discussed until agreement.

## 3 Results

### 3.1 Descriptive Synthesis

#### 3.1.1 Study Selection and Characteristics

Figure 1 displays the latest search yield and each stage of the search in a PRISMA flow chart. The search discovered 13 503 potential articles across five electronic databases. Following removal of duplicates and title and abstract screening, 91 articles were read in full, with a further 46 excluded due to failure to meet the inclusion criteria. Sixteen additional studies were identified through citation searching of all included full texts and existing narrative reviews within the area [8, 57, 58]. Therefore, 61 published reports containing 63 experiments were included in the qualitative and descriptive analysis (henceforth terms studies, trials or experiments encompass all 63 included protocols; experiments from the same journal article are differentiated by Experiment 1 and 2 corresponding to the order presented within original articles [13, 16]). Forty‐two experiments were randomized, 26 of which used a repeated measures or crossover design, with the remaining 11 and five parallel groups or mixed study designs, respectively. The remaining 21 trials were not randomized (17 repeated measures, two parallel groups and two mixed design). Only eight experiments incorporated blinding, as three studies blinded participants only [27, 28, 30] and five were double‐blinded [45, 53, 59, 60, 61]. Study and participant characteristics are reported in Table 1.

**FIGURE 1:**
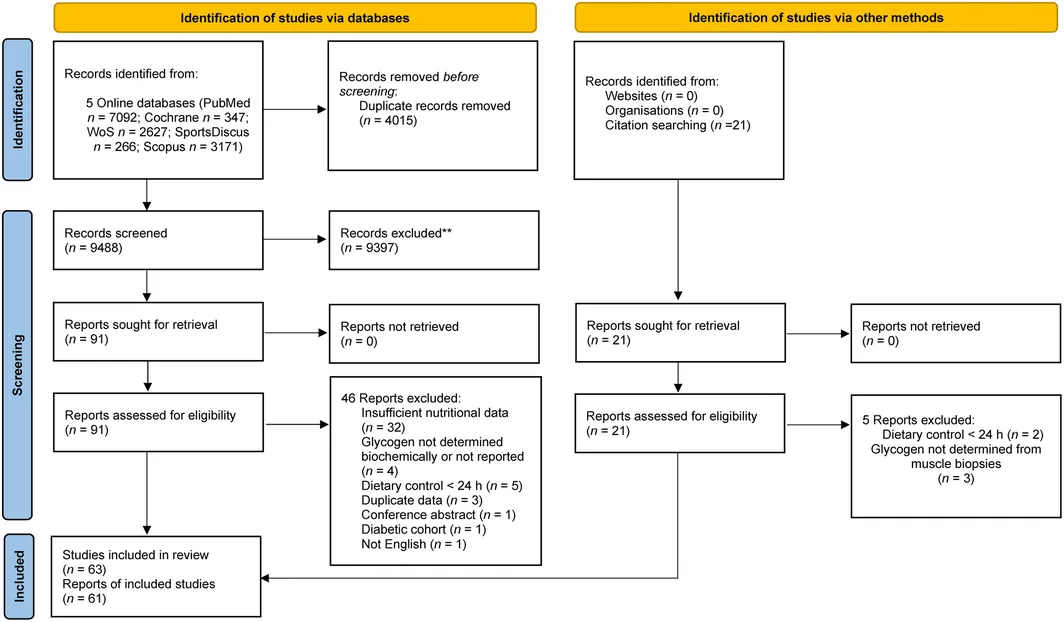
PRISMA flowchart displaying each stage of the literature search conducted to attain articles included within the current systematic review and meta‐analysis.

**TABLE 1:** Summary of studies included within systematic review of CHO loading literature (*n* = 63).

Study	Experimental condition	Participants		Glycogen depletion exercise	CHO intake		Duration	Biopsy location	Whole muscle glycogen (mmol·kg^−1^ DM)	Significance between conditions
		*n* and sex	Training status		Relative (g·kg^−1^·day^−1^)	Absolute (g·day^−1^)				
Adamo et al. [25]	LCHO	9 M	3	Cycling	3.4	262	48 h	VL	294.0	***p* < 0.05** between conditions
	vHCHO				8.2	640			480.0	
Akermark et al. [62]	MCHO	16 M	5	Other	6.2	498	72 h	VL	352.4 ± 30.5	***p* < 0.05** between conditions
	HCHO	16 M	5		8.4	675			430.7 ± 30.5	
Arkinstall et al. [2]	LCHO	7 M	3	Cycling	0.7	56	48 h	VL	180.0 ± 17.0	***p* < 0.001** between low and very high CHO conditions
	LCHO				0.7	56			223.0 ± 25.0	
	vHCHO				10.0	803			591.0 ± 34.0	
	vHCHO				10.0	803			601.0 ± 52.0	
Arnall et al. [26]	vHCHO +3d	6 M	3	Cycling	9.1	720	72 h	VL	752.6 ± 130.5	***p* < 0.05** vs. baseline
	vHCHO +5d	6 M	3		9.1	720			791.7 ± 60.9	
	vHCHO +7d (+ days investigated ability to maintain enhanced stores)	5 M	3		9.1	720			596 ± 43.5	
Bergström et al. [3]	High fat + PRO	9 M	3	Cycling	0.0	0	72 h	VL	152.2 ± 24.2	***p* < 0.001** between conditions
	HCHO				8.3	575			799.4 ± 72.5	
Blom et al. [13] Experiment 1	HCHO untrained	6 M	1	Running	7.9	600	70 h	G	287.1 ± 37.8	***p* < 0.05** between conditions
	HCHO well trained	6 M	4		9.0	600			583.3 ± 132.7	
Blom et al. [13] Experiment 2	HCHO (exercise)	6 M	3	Running	8.3	600	70 h	G	654.2 ± 94.8	*p* > 0.05 between conditions
	HCHO (rest) (days pre‐depletion)				8.3	600			711.7 ± 104.4	
Bosch et al. [63]	MCHO	7 M	2	No	4.0	303	72 h	VL	539.4 ± 30.5	NC
	HCHO	9 M	2		8.1	600			843.9 ± 17.4	
Bradley et al. [40]	MCHO	7 M	5	Other	3.0	264	36 h	VL	449.0 ± 19.3^a^	*Unclear* differences between conditions (magnitude‐based statistics)
	MCHO	7 M	5		6.0	528			444.0 ± 30.6^a^	
Bradley et al. [27]	MCHO (immediate)	8 M	2	Cycling	6.0	524	72 h	VL	416 ± 56.9^a^	NC
	MCHO (delayed) (acute post‐depletion refeed strategy)	7 M	2		6.0	524			362 ± 54.0^a^	
Burke et al. [64]	vHCHO low GI	5 M	4	Cycling	10.0	687	24 h	VL	462.8 ± 33.5	***p* < 0.05** between conditions
	vHCHO high GI				10.0	687			575.9 ± 32.6	
Burke et al. [65]	HCHO	8 M	3	Cycling	7.0	518	24 h	VL	522.9 ± 41.8	*p* > 0.05 between conditions
	HCHO + fat +PRO				7.0	518			452.4 ± 16.5	
	vHCHO Energy match				11.8	873			519.4 ± 25.2	
Burke et al. [66]	vHCHO Gorging	8 M	3	Cycling	10.0	702	24 h	VL	565.1 ± 44.4	*p* > 0.05 between conditions
	vHCHO Nibbling				10.0	702			628.6 ± 53.1	
Burke et al. [59]	MCHO	7 M	4	Cycling	5.8	419	72 h	VL	485.0 ± 48.4^a^	***p* < 0.05** between conditions
	vHCHO				9.0	646			572 ± 40.4^a^	
Bussau et al. [9]	Baseline	8 M	4	No	5.8	447	24 h	VL	417.6 ± 17.4	***p* < 0.05** vs. baseline
	vHCHO				10.2	785	72 h		848.3 ± 74.0	
Costill et al. [16] Experiment 1	HCHO complex	6 M	3	Running	9.0 + 5.8	648 + 415	24 h + 24 h (48 h total)	G	725.1 ± 41.3 642.1 ± 32.6	***p* < 0.05** between conditions
	HCHO simple				9.0 + 5.8	648 + 415				
Costill et al. [16] Experiment 2	LCHO	4 M	3	Running	2.4	188	24 h	G	289.7 ± 33.9	***p* < 0.05** between high conditions vs. LCHO and MCHO
	MCHO				4.7	375			322.8 ± 17.0	
	HCHO				6.6	525			440.2 ± 90.9	
	HCHO				6.6	525			546.4 ± 47.4	
Costill et al. [44]	HCHO control	8 M	3	Cycling	6.6	500	48 h	VL	604.2	*p* > 0.05 between conditions
	HCHO distal (multiple biopsies)	(4 M)			6.6	500			551.1	
De Bock et al. [67]	MCHO	8 M	2	No	6.1	458	72 h	VL	455.0 ± 40.0	*p* > 0.05 between conditions
	MCHO				6.1	458			466.0 ± 19.0	
Doering et al. [68]	Baseline	6 M 1 F	3	No	5.2	396	24 h	VL	583.6 ± 45.3^a^	Conditions vs. baseline ***p* = 0.04** ***p* = 0.01**
	vHCHO			Cycling	10.6	807	96 h		835.1 ± 46.1^a^	
	vHCHO 2nd depletion			Cycling	10.6	807	96 h		848.3 ± 45.5^a^	
Duhamel et al. [32]	LCHO	10 M	1	Cycling	1.1	94	96 h	VL	365.7 ± 26.6	***p* < 0.05** between conditions
	HCHO				7.5	657			568.5 ± 44.9	
Fairchild et al. [39]	Baseline	7 M	3	No	~5.6	~430	96 h	VL	474.6 ± 35.7	***p* < 0.05** vs. baseline
	vHCHO			Cycling	10.3	793	24 h		862.2 ± 57.0	
Fell et al. [41]	vHCHO	8 M	3		12.0	883	36 h	VL	698.0 ± 34.6^a^	*p* = 0.14 between conditions
	vHCHO			Cycling	12.0	883			742.0 ± 38.5^a^	
	vHCHO				12.0	883			767.0 ± 30.8^a^	
Flynn et al. [28]	HCHO	8 M	3	Cycling	6.9	500	48 h	VL	794.3 ± 28.7	*p* > 0.05 between conditions
	HCHO				6.9	500			778.7 ± 47.4	
	HCHO				6.9	500			824.3 ± 41.8	
	HCHO				6.9	500			836.1 ± 65.3	
Fogelholm et al. [37]	vHCHO half marathon	6 M	4	Running	9.1	598	96 h	VL	281.0 ± 25.4^a^	*p* > 0.05 between conditions
	vHCHO Fartlek				9.5	626			343.5 ± 27.6^a^	
Foskett et al. [60]	vHCHO	6 M	2	Running	10.0	750	48 h	VL	512.0 ± 41.6^a^	*p* > 0.05 between conditions
	vHCHO				10.0	750			533.0 ± 31.4^a^	
Galbo et al. [29]	Fat + PRO	7 M	3	Cycle + run	0.0	0	96 h	VL	195.5 ± 26.1	***p* < 0.05** between conditions
	HCHO				7.3	559			487.1 ± 65.2	
Goforth et al. [69]	vHCHO	14 M	2	Cycling	9.3	720	72 h	VL	729.0 ± 83.9^a^	***p* < 0.05** vs. baseline
Greiwe et al. [18]	vHCHO untrained	2 M 4 F	1	Cycling	10.0	630	48 h	VL	428.5 ± 85.7	***p* < 0.05** between conditions
	vHCHO 10wk trained		2		10.0	630			795.6 ± 124.9	
Hawley et al. [42]	MCHO	6 M	4	No	5.9	426	72 h	VL	459.0 ± 33.9^a^	***p* < 0.05** between conditions
	vHCHO				9.3	661			565.0 ± 25.3^a^	
Helge et al. [70]	7wk high Fat +1wk HCHO	7 M	2	Cycling	6.9	595	7 d	VL	872.0 ± 59.0	***p* < 0.05** between conditions
	8wk HCHO	6 M	2		7.0	599			688.0 ± 43.0	
Hickner et al. [17]	vHCHO trained	3 M	3	Cycling	10.0	736	48 h	VL	635.1 ± 167.5	***p* < 0.05** between trained vs. untrained *p* = 0.46 for 48 vs. 72 h trained ***p* < 0.01** for 48 vs. 72 h untrained
	vHCHO trained	3 M			10.0		72 h		792.1 ± 90.5	
	vHCHO untrained	3 M	1		10.0	688	48 h		275.8 ± 31.3	
	vHCHO untrained	3 M			10.0		72 h		584.6 ± 37.4	
Hill et al. [30]	vHCHO + PRO	6 M	3	No	8.0 + 10.0	592 + 740	14 d + 48 h	VL	633.0 ± 82.0	*p* > 0.05 between conditions
	vHCHO				8.0 + 10.0	592 + 740			695.0 ± 61.0	
Impey et al. [31]	MCHO for all, post‐load running exercise manipulated	11 M	2	Running	6.0	457	48 h	VL	446.0 ± 74.0	***p* < 0.001** for males vs. females in G *p* > 0.05 between all other conditions.
					6.0	457		VL	421.0 ± 74.0	
					6.0	457		VL	409.0 ± 91.0	
					6.0	457		G	504.0 ± 157.0	
					6.0	457		G	498.0 ± 207.0	
					6.0	457		G	542.0 ± 117.0	
		10 F	2		6.0	369		VL	367.0 ± 148.0	
					6.0	369		VL	430.0 ± 128.0	
					6.0	369		VL	373.0 ± 95.0	
					6.0	369		G	433.0 ± 117.0	
					6.0	369		G	296.0 ± 117.0	
					6.0	369		G	438.0 ± 140.0	
James et al. [38]	vHCHO	6 M	3	Cycling	10.5	872	72 h	VL	796.1 ± 44.4^a^	*p* > 0.05 between conditions ***p* < 0.05** vs. baseline for all
	vHCHO pre‐menses	6 F	3		9.9	684			878.7 ± 35.5^a^	
	vHCHO post‐menses				9.9	684			839.6 ± 24.9^a^	
Jansson and Kaijser, [71]	LCHO	4 M 3 F	2	No	0.3	21	120 h	VL	239.0 ± 25.7^a^	***p* < 0.05** between conditions
	MCHO				5.9	383			445.0 ± 43.5^a^	
Jensen et al. [24]	LCHO	11 M	3	Cycling	0.2	15	72 h	VL	257 (233–360)	***p* < 0.001** vs. LCHO only
	MCHO				4.7	361	24 h		488 (460–532)	
	HCHO				8.0	616	72 h		499 (472–568)	
									*Median (IQR)*	
Jones et al. [61]	HCHO	10 M	2	Cycling	9.0	654	36 h	VL	355.0 ± 20.6^a^	***p* < 0.05** between conditions
	HCHO + Blackcurrant supplement				9.0	654	36 h		493.0 ± 26.9^a^	
Jones et al. [51]	MCHO	11 M	3	Cycling	5.9	419	48 h	VL	460.9 ± 30.5^a^	***p* < 0.05** vs. vHCHO
	HCHO				7.8	560	48 h		506.1 ± 37.4^a^	
	vHCHO				9.7	691	48 h		635.5 ± 23.5^a^	
Kavouras et al. [14]	LCHO	12 M	3	Cycling	1.4	100	72 h	VL	314.1 ± 24.4	***p* < 0.05** between conditions
	vHCHO				8.2	600			454.6 ± 40.9	
Kirwan et al. [72]	MCHO + HCHO	10 M	4	Running	6.2 + 8.0	413 + 533	72 h + 96 h	G	524.6 ± 30.9	***p* < 0.05** between conditions
	MCHO + MCHO				6.2 + 3.9	413 + 260	72 h + 96 h		355.0 ± 41.8	
Kochan et al. [33]	vHCHO no depletion	6 M	1	No	11.3	823	96 h	VL	502.3 ± 67.6	***p* < 0.05** between conditions
	vHCHO depletion (one leg model)			Cycling	11.3	823			912.9 ± 77.3	
Lamb et al. [73]	vHCHO pasta	7 M	4	Running	11.3	809	84 h	VL	566.4 ± 55.2	*p* > 0.05 between conditions ***p* < 0.05** vs. baseline for both
	vHCHO supplement	7 M	4		11.7	800			653.4 ± 51.3	
MacDougall et al. [74]	MCHO	3 M	2	Cycling	3.2	252	24 h	VL	347.6 ± 10.0	*p* > 0.05 between conditions
	HCHO	3 M	2		7.7	617			367.6 ± 17.4	
Maunder et al. [75]	LCHO	13 M	2	Running	2.4	169	72 h	VL	236.2 ± 38.2^a^	***p* < 0.05** vs. LCHO only
	MCHO				5.0	322			360.2 ± 27.2^a^	
	MCHO				6.5	460			355.0 ± 26.1^a^	
McInerney et al. [46]	Baseline	6 M	3	No	6.0	435	24 h	VL	435.0 ± 57.0	***p* < 0.01** between conditions and vs. baseline
	vHCHO			Cycling	12.0	870	48 h		713.0 ± 60.0	
	vHCHO 2nd depletion			Cycling	12.0	870			409.0 ± 40.0	
McLay et al. [15]	MCHO midfollicular	9 F	3	Cycling	5.2	359	72 h	VL	575.0 ± 47.7^a^	***p* = 0.02** for moderate CHO vs. all other conditions
	MCHO midluteal				5.2	359			771.0 ± 45.7^a^	
	HCHO midfollicular				8.4	551			728.0 ± 47.0^a^	
	HCHO midluteal				8.4	551			756.0 ± 37.3^a^	
Nelson et al. [76]	HCHO depletion	12 M	2	Cycling	6.6	NC	72 h	VL	602.0 ± 39.5^a^	***p* < 0.05** vs. baseline *p* > 0.05 between conditions
	HCHO no depletion (one leg model)			No	6.6	NC			488.0 ± 29.4^a^	
Nicklas et al. [77]	MCHO midluteal	6 F	2	Cycling	3.9	237	72 h	VL	451.1 ± 23.9	*p* > 0.05 between conditions
	MCHO midfollicular				4.2	254			404.6 ± 25.7	
Palmer et al. [78]	MCHO steady state	6 M	4	Cycling	5.4	398	72 h	VL	678.6 ± 60.9	*p* > 0.05 between conditions
	MCHO variable intensity exercise				5.4	398			643.8 ± 100.1	
Rauch et al. [49]	MCHO	8 M	4	No	6.2	439	72 h	VL	454.0 ± 34.8	***p* < 0.0001** between conditions
	vHCHO				10.5	750			664.5 ± 39.2	
Roberts et al. [34]	HCHO	7 M	2	Cycling	8.1	637	6 days	VL	789.0 ± 38.0	***p* < 0.01** between conditions
	HCHO + Creatine	7 M			8.2	645			948.0 ± 75.0	
Sasaki et al. [79]	MCHO	5 M	2	Cycling	3.4	214	7 days	VL	260.8 ± 45.9	***p* < 0.05** between conditions
	HCHO				8.0	499			840.2 ± 94.4	
Schytz et al. [52]	MCHO	22 M	2	Cycling	4.0	309	48 h	VL	367.0 ± 14.1^a^	***p* < 0.0001** between conditions
	HCHO				10.0	773			525.0 ± 14.4^a^	
Sherman et al. [10]	MCHO	6 M	4	Running	5.1	353	72 h	G	693.4 ± 28.7	***p* < 0.05** between MCHO and HCHO conditions
	3d LCHO 3d HCHO				7.8	542			903.9 ± 50.9	
	3d MCHO 3d HCHO				7.8	542			884.4 ± 32.6	
Sherman et al. [35]	HCHO exercise	5 M	4	Running	12.7 + 7.1	800 + 450	24 h+	G	474.6 ± 30.5	***p* < 0.05** for both conditions vs. pre‐marathon
	HCHO rest	5 M			11.6 + 6.5	800 + 450	6 days		613.8 ± 26.1	
	Pre‐marathon	(10 M)			8.2	542	72 h		852.6 ± 33.1	
Tarnopolsky et al. [36]	MCHO	6 F	4	No	5.4	317	48 h	VL	438.0 ± 105.6	*p* > 0.05 between conditions
	HCHO	6 M	4		7.1	478			471.4 ± 28.3	
Tarnopolsky et al. [54]	MCHO	8 F	3	No	4.8	274	96 h	VL	407.3 ± 27.6^a^	***p* < 0.01** between male HCHO vs. male LCHO
	MCHO				6.4	370			407.8 ± 20.2^a^	
	HCHO	7 M	3		6.6	492			401.5 ± 29.0^a^	
	HCHO				8.2	614			565.4 ± 33.3^a^	
Tarnopolsky et al. [53]	HCHO	6 M	3	Cycling	7.9	585	96 h	VL	660.2 ± 49.0^a^	***p* < 0.05** for males vHCHO and HCHO vs. MCHO ***p* < 0.05** for females HCHO vs. MCHO (habitual)
	vHCHO				10.5	778			741.1 ± 76.6^a^	
	MCHO (habitual)				6.1	452			537.2 ± 32.1^a^	
	MCHO	7 F	3		6.4	380			650.6 ± 71.0^a^	
	HCHO				8.8	523			737.7 ± 74.9^a^	
	MCHO (habitual)				5.1	303			629.1 ± 87.3^a^	
Tomcik et al. [45]	MCHO + Creatine	9 M	4	Cycling	6.0	469	48 h	VL	589.0 ± 31.3^a^	***p* < 0.05** between MCHO and vHCHO conditions
	vHCHO + Creatine				12.0	938			692.0 ± 32.3^a^	
	MCHO	9 M			6.0	469			581.0 ± 42.3^a^	
	vHCHO				12.0	938			724.0 ± 36.0^a^	
Vandenberghe et al. [80]	MCHO	10 NC	2	Cycling	4.6	313	5 days	VL	364.0 ± 23.0	***p* < 0.05** between conditions
	HCHO				7.7	525	72 h		568.0 ± 35.0	
Walker et al. [50]	MCHO	6 F	4	Cycling	4.7	266	96 h	VL	625.2 ± 50.1	***p* < 0.05** between conditions
	HCHO				8.2	464			709.0 ± 44.8	
Widrick et al. [81]	LCHO	8 M	3	Cycling	2.3	181	48 h	VL	434.1 ± 26.1	***p* < 0.05** between LCHO and MCHO conditions
	LCHO				2.3	181			477.2 ± 23.1	
	MCHO				6.5	511			783.9 ± 42.2	
	MCHO				6.5	511			740.4 ± 45.2	

*Note:* Included studies reported exercise status and CHO intake for at least 24 h pre‐muscle biopsy. Table outlines experimental conditions, participant characteristics, CHO loading interventions (CHO intake [relative and absolute] and duration), muscle biopsy location and post‐load muscle glycogen concentration. Data presented as means ± SE unless otherwise stated. LCHO, MCHO, HCHO, and vHCHO for low, moderate, high, and very high carbohydrate intake as previously defined (< 3, 3–6.5, > 6.5–9, and > 9 g·kg^−1^·day^−1^ respectively). *p* values indicate significance between conditions with bold values the threshold for significance < 0.05.

Abbreviations: F, females; G, *gastrocnemius*; h, hours; M, males; NC, not confirmed; PRO, protein; VL, *vastus lateralis*.

^a^ Indicates calculated SE from SD presented in original article. Training status was classified according to De Pauw et al. [21] and Decroix et al. [22] with 1, 2, 3, 4, and 5 corresponding to sedentary, recreationally active, endurance trained, well trained and elite, respectively.

#### 3.1.2 Participants

The descriptive synthesis included 641 participants in total, primarily aged 18–30 years (83% of experiments), with only one study reporting participant mean age > 35 years old (37 ± 7 years; [68]). Only 5% of experiments included solely female participants [15, 50, 77] whilst 16% recruited both males and females either as combined [18, 51, 68, 71, 80] or separate groups [31, 36, 38, 53, 54], with the remaining 79% including males exclusively. In total, only 74 females were included across all trials (12% of participants). Participants were primarily endurance trained or well trained as 40% and 22% of trials recruited participants at performance levels 3 and 4, respectively [21, 22]. Twenty‐seven percent recruited recreationally active individuals, whilst the remaining 13% had sedentary participants [32, 33], elite team sport athletes from rugby and ice hockey [40, 62], or compared untrained vs. recreationally active, trained or well‐trained participants [13, 17, 18] (Experiment 1).

#### 3.1.3 CHO Loading Interventions (Dose, Duration, and Type)

Twenty‐six studies compared experimental conditions of different defined levels of CHO intake (2 compared low vs. moderate [71, 81], 4 low vs. high [3, 14, 29, 32], 1 compared low vs. very high [2], 11 moderate vs. high [10, 15, 25, 36, 50, 59, 62, 63, 74, 79, 80], 7 compared moderate vs. very high [9, 42, 45, 46, 49, 52, 68] and 1 compared high vs. very high CHO [65]). A further eight studies [13, 34, 37, 38, 40, 70, 73, 77] (Experiment 1) compared different CHO intakes, however expressed relative to body mass (g·kg^−1^·day^−1^) conditions fell within the same defined category (i.e., moderate). Six trials included multiple conditions of varied CHO intake in a dose–response type study design [16, 24, 51, 53, 54, 75] (Experiment 2). Twenty‐one studies compared the exact same relative CHO intake across conditions, with intakes of 6–7 [27, 28, 31, 44, 67, 76] and 9–10 g·kg^−1^·day^−1^ [17, 18, 26, 60, 61, 64, 66, 69] most common. Manipulation of CHO intake/CHO loading was not an aim of these studies, but rather a controlled factor. Trials were included within descriptive synthesis as all inclusion criteria were met and information was relevant for between‐study comparisons, provided the primary study intervention did not significantly impact glycogen concentrations (if this was the case, only the control conditions were considered).

The most common CHO loading/dietary control durations were 48 and 72 h (13 and 21 trials, respectively; Table 1). Six and seven trials used 24 and 96 h loading periods, respectively, whilst others used less common durations of 36, 70, or 84 h. A further five experiments had periods ≥ 5 days [34, 50, 70, 71, 72] whilst five studies used different periods of dietary control between conditions, where a control/baseline period of moderate CHO intake and a muscle biopsy was followed by experimental conditions [35, 46, 68, 80].

CHO type was poorly described, as 28 out of 63 trials did not provide enough information regarding CHO consumed to judge glycaemic index. Twenty‐eight studies provided mixed diets containing both high and low glycaemic index foods, whilst five trials provided exclusively high glycaemic index CHO sources [9, 29, 39, 65, 66]. Only two trials directly compared different types of CHO sources [16, 64] (Experiment 1).

Overall, dietary interventions were well controlled, as 84% of studies provided participants with food items and meals. However, only 10% of these studies measured and reported actual dietary compliance. The remaining nine experiments (16%) either did not specifically state how dietary intake was controlled [16, 33, 35, 63, 71] (Experiment 1 and 2) or provided participants with meal plans [28, 37, 42, 77].

#### 3.1.4 Exercise Interventions

Exercise pre‐loading was very common (50 out of 63 studies), with some studies allowing participants to continue habitual training, which was standardized between conditions [40, 59, 62, 70, 72, 78], however, most trials utilized specific glycogen depleting exercise (44 out of 48 studies). Cycling was the primary exercise mode as 72% of protocols were in cycling models. The remaining 22% and 6% of studies were in running or other mixed protocols, respectively. Only 22 studies allowed exercise during the loading period (35%); these sessions were primarily light‐to‐moderate‐intensity steady state, short duration sessions (≤ 60 min) designed to mimic a pre‐competition taper or described as “habitual” training. In the remaining 65%, participants were rested throughout the loading period.

### 3.2 Exploratory Linear Regression

Recreationally active individuals demonstrated a significant linear relationship between relative CHO intake and whole muscle glycogen (*F*_1,42_ = 20.91, *r* ^2^ = 0.332, *p* < 0.001; Figure 2a). The strength of the linear relationship increased in endurance trained individuals (*F*_1,75_ = 72.14, *r* ^2^ = 0.490, *p* < 0.001; Figure 2b), with results consistent across second‐degree polynomial models (*r* ^2^ = 0.332, *p* < 0.001 and *r* ^2^ = 0.515, *p* < 0.001, for recreationally active and endurance trained, respectively). In well‐trained participants, simple linear regression indicated that relative CHO intake only predicted 8.6% of the variation in muscle glycogen (*F*_1,24_ = 2.24, *r* ^2^ = 0.086, *p* = 0.147; Figure 2c), which was also consistent in a second‐degree polynomial model (*r* ^2^ = 0.092, *p* = 0.331; Figure 2f).

**FIGURE 2:**
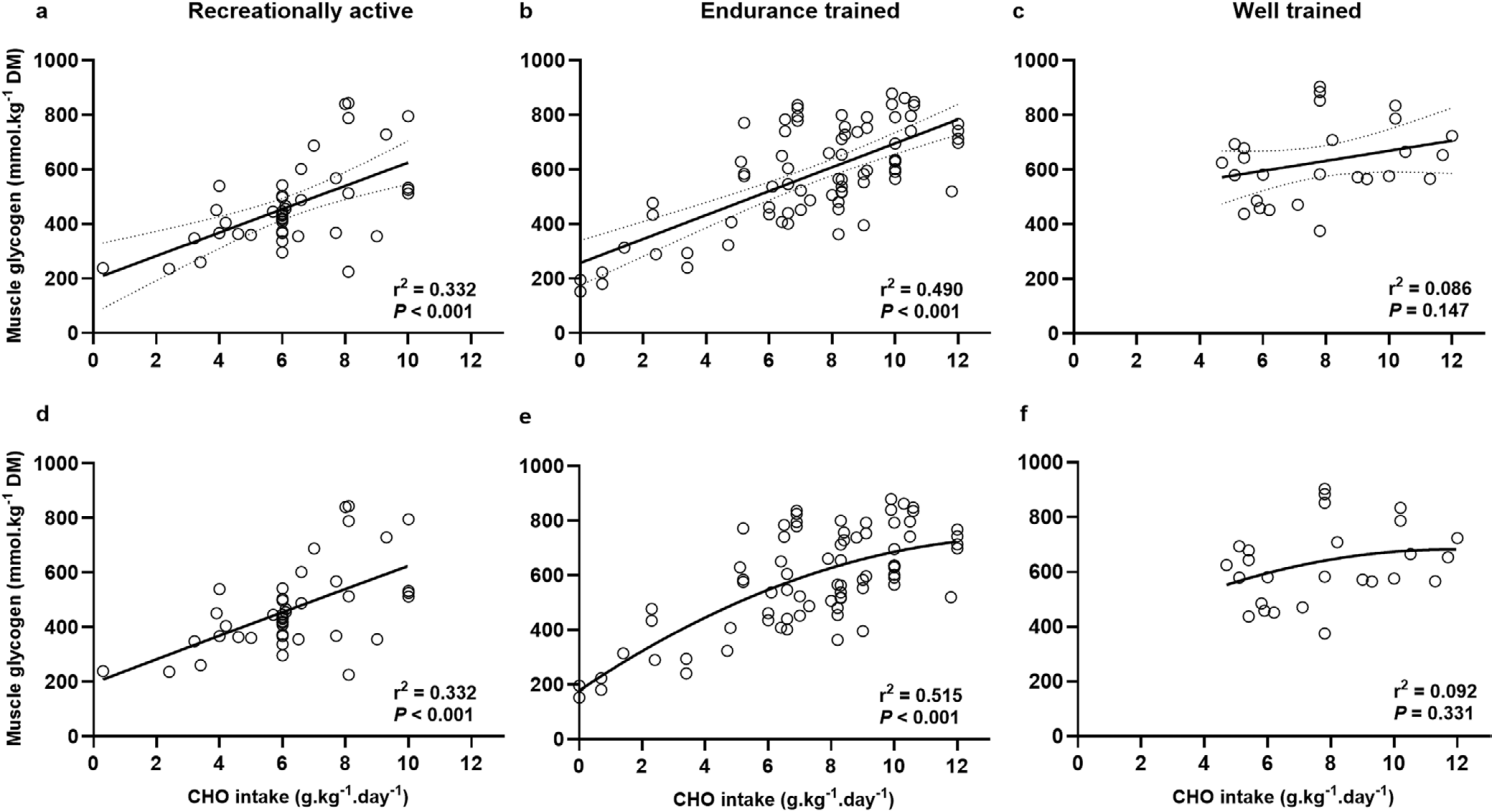
Exploratory simple linear (a–c) and nonlinear second‐degree polynomial regression analyses (d–f) of relative CHO intake and skeletal muscle glycogen in recreationally active (a, d), endurance trained (b, e), and well‐trained individuals (c, f). Each data point represents a study group/condition. Included study groups reported exercise status and CHO intake for at least 24 h pre‐muscle biopsy, and studies which implemented further interventions which may have impacted glycogen concentrations were excluded. Dotted lines display 95% confidence interval bands.

Regression results were consistent for absolute CHO intake, as recreationally active (*F*_1,40_ = 16.14, *r* ^2^ = 0.288, *p* < 0.001; Figure 3a) and endurance trained individuals (*F*_1,75_ = 65.02, *r* ^2^ = 0.464, *p* < 0.001; Figure 3b) showed a significant linear relationship between absolute CHO intake and muscle glycogen. Results were consistent across second‐degree polynomial regression models (*r* ^2^ = 0.297, *p* < 0.001 and *r* ^2^ = 0.497, *p* < 0.001, respectively). For well‐trained participants, absolute CHO intake only predicted 4.7% of the variance in glycogen concentration (*F*_1,23_ = 1.13, *p* = 0.298; Figure 3c), which was maintained in a second‐degree polynomial model (*r* ^2^ = 0.047, *p* = 0.587; Figure 3f).

**FIGURE 3:**
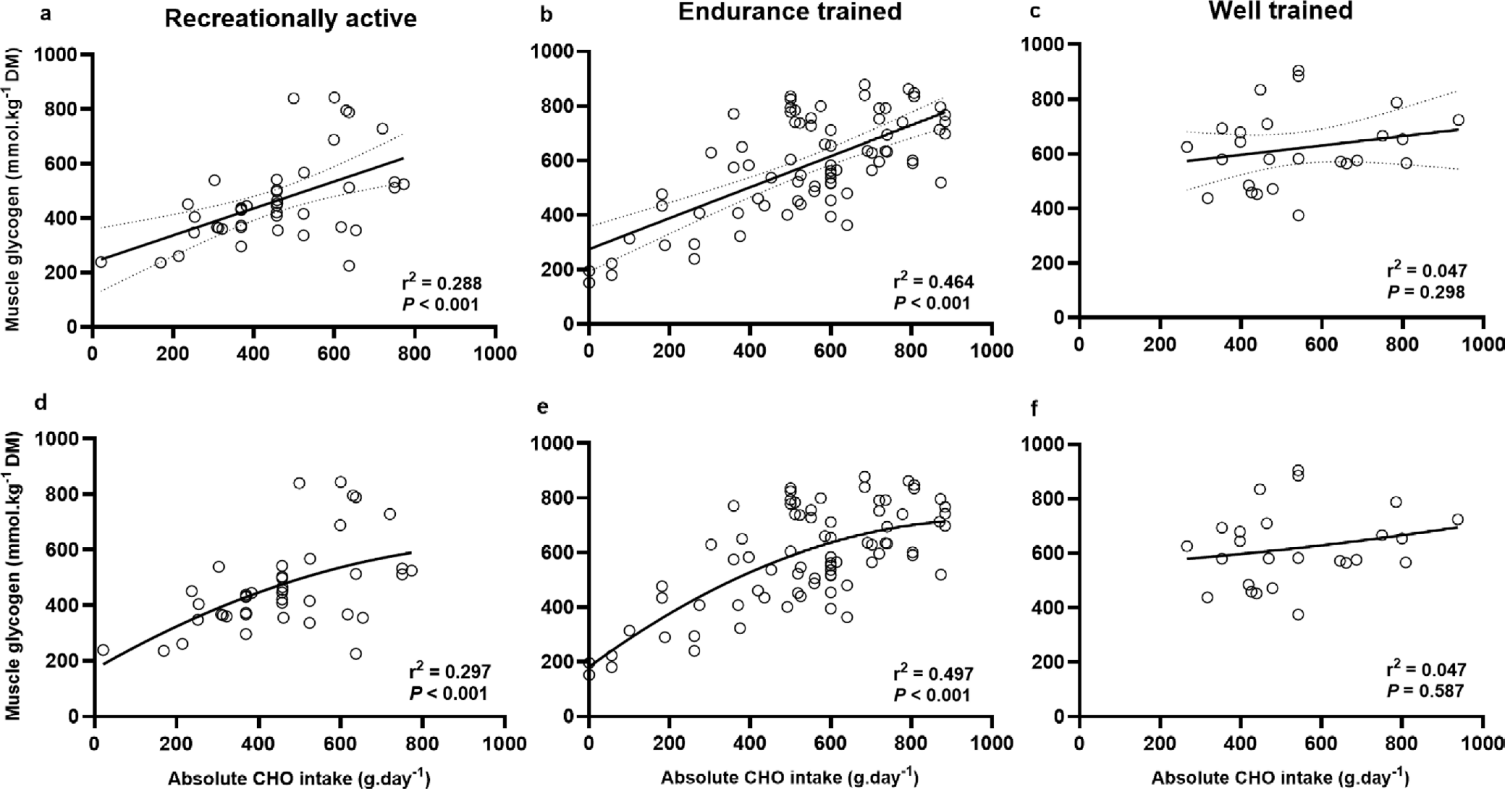
Exploratory simple linear (a–c) and nonlinear second‐degree polynomial regression analyses (d–f) of absolute CHO intake and skeletal muscle glycogen in recreationally active (a, d), endurance trained (b, e) and well‐trained individuals (c, f). Each data point represents a study group/condition. Included study groups reported exercise status and CHO intake for at least 24 h pre‐muscle biopsy, and studies that implemented further interventions which may have impacted glycogen concentrations were excluded. 95% confidence interval bands are displayed as dotted lines.

### 3.3 Effectors of Muscle Glycogen Concentration

#### 3.3.1 CHO Intake

Eighteen studies were included within the meta‐analyses (13 repeated measures and 5 parallel groups; Figure 4). Increasing relative CHO intake from a low‐moderate (< 6.5 g·kg^−1^·day^−1^) to high‐very high intake (> 6.5 g·kg^−1^·day^−1^) increased muscle glycogen by 191.9 mmol·kg^−1^ DM (95% CI = 142.8 to 241.1 mmol·kg^−1^ DM, *n* = 111, *Z* = 7.66, *p* < 0.0001) and by 107.6 mmol·kg^−1^ DM (95% CI = −10.5 to 225.7 mmol·kg^−1^ DM, *n* = 81, *Z* = 1.79, *p* = 0.07) for repeated measures and parallel group trials, respectively. There was considerable and statistically significant heterogeneity for both repeated measures (Chi^2^ = 271.78, df = 16, *p* < 0.0001, *I* ^2^ = 94%) and parallel groups designs (Chi^2^ = 49.87, df = 4, *p* < 0.0001, *I* ^2^ = 92%), which was expected at this stage of the analysis, as sources of heterogeneity were yet to be explored.

**FIGURE 4:**
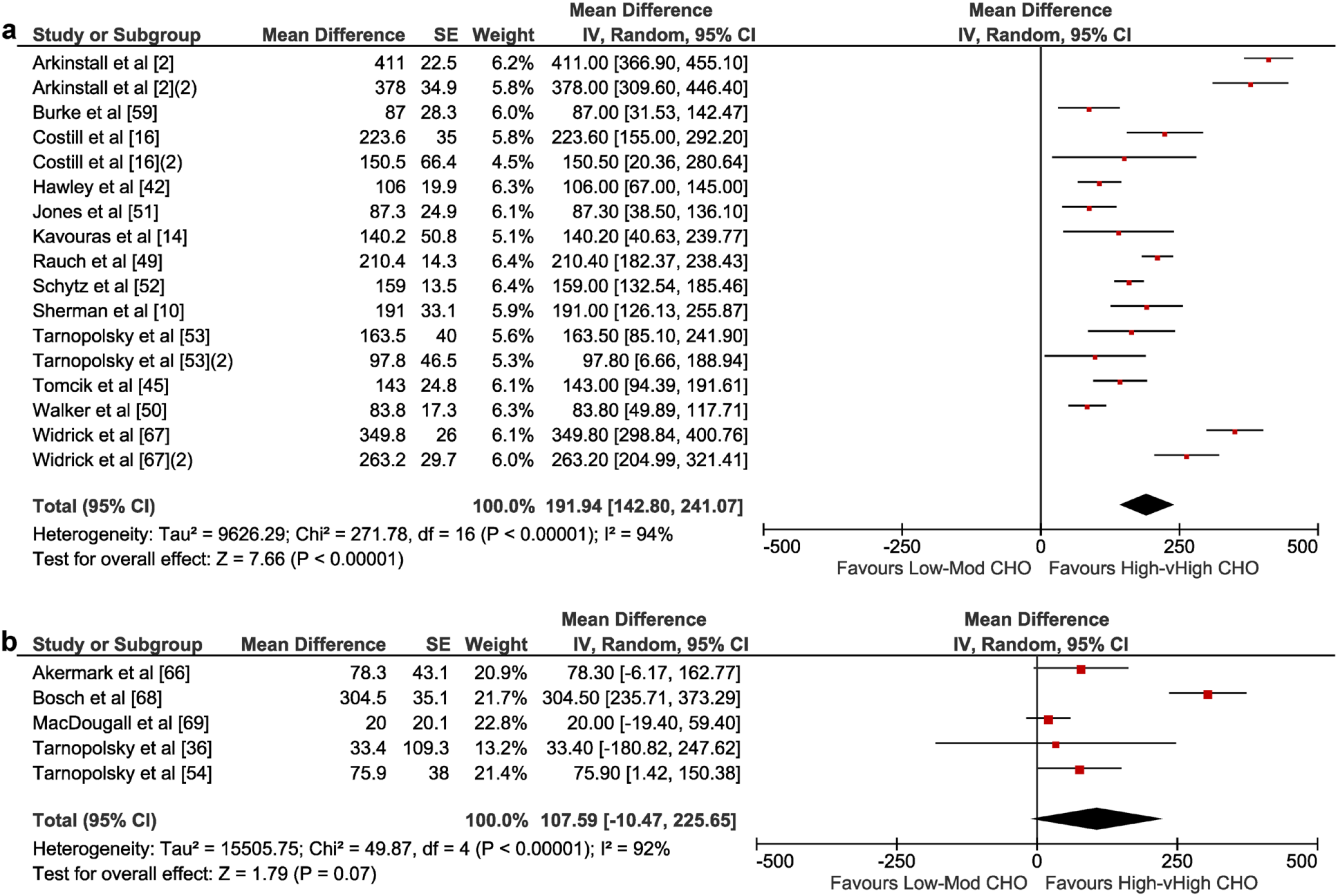
Forest plot of general inverse‐variance random‐effects meta‐analyses conducted for randomized crossover (a) and parallel groups (b) study designs that investigated the effects of a low‐moderate versus high‐very high CHO intake (Low‐MCHO < 6.5 g·kg^−1^·day^−1^ vs. high‐vHCHO > 6.5 g·kg^−1^·day^−1^) on whole skeletal muscle glycogen obtained from muscle biopsy samples. Included study groups reported exercise status and CHO intake for at least 24 h pre‐muscle biopsy.

The Δ relative CHO intake between low‐moderate versus high‐very high conditions was assessed as incremental 1 g·kg^−1^·day^−1^ subgroups from 1.5 to > 5.5 g·kg^−1^·day^−1^, however the 4.5–5.5 g·kg^−1^·day^−1^ subgroup was not included due to only containing one pairwise comparison [52]. There was a significant quantitative subgroup effect (*p* = 0.01), where increased Δ between relative CHO intakes resulted in an increase in muscle glycogen content across subgroups (Figure 5). However, effect estimates should be interpreted with caution, as despite an improvement in heterogeneity, all subgroups still displayed significant heterogeneity (*I* ^2^ = 93%, *p* = 0.0001; *I* ^2^ = 54%, *p* = 0.06; *I* ^2^ = 88%, *p* < 0.0001; *I* ^2^ = 96%, *p* < 0.0001, for Δ relative CHO intakes of 1.5–2.5, 2.5–3.5, 3.5–4.5 and > 5.5 g·kg^−1^·day^−1^, respectively). Covariate distribution was similar for most subgroups (apart from 1.5–2.5 g·kg^−1^·day^−1^), as 4–6 study pairwise comparisons contributed to each subgroup with a similar number of participants (*n* = 28–38 participants per condition, per subgroup; Figure 5).

**FIGURE 5:**
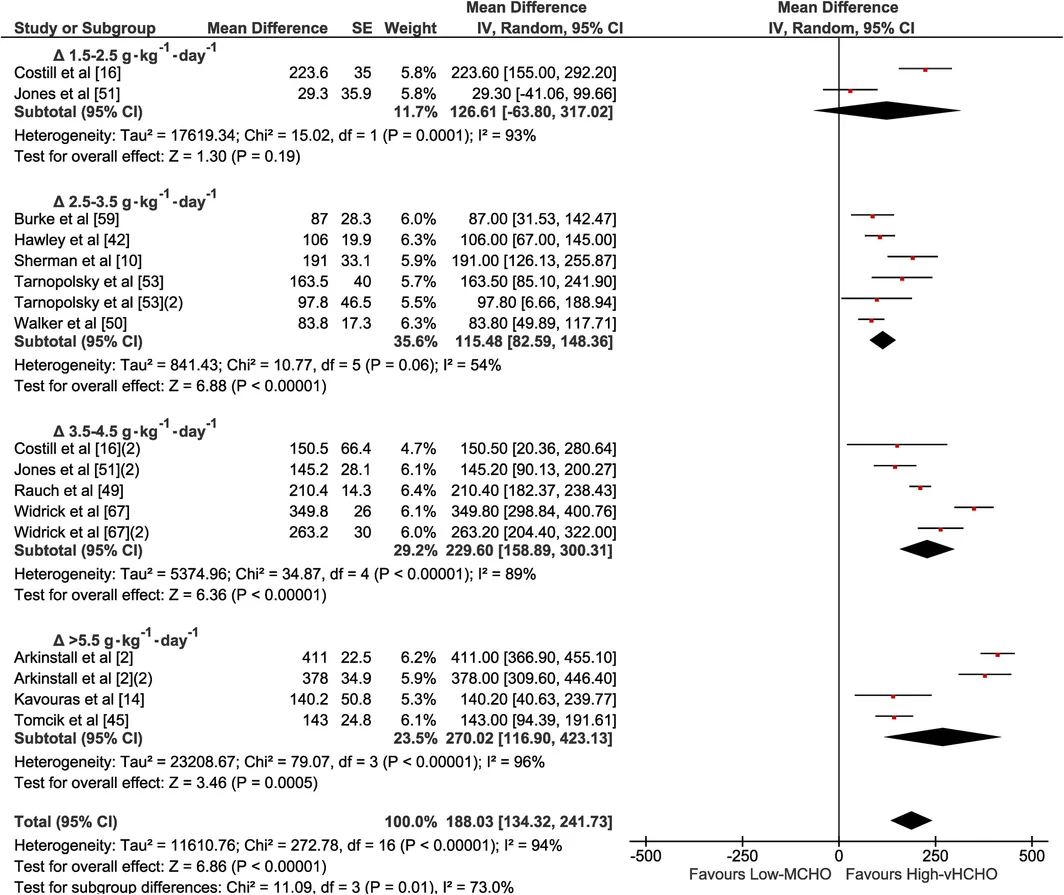
Forest plot of general inverse‐variance random‐effects meta‐analysis conducted in crossover design studies with subgroups for the Δ relative CHO intake between defined low‐moderate and high‐very high CHO conditions (Low‐MCHO < 6.5 g·kg^−1^·day^−1^ vs. high‐vHCHO > 6.5 g·kg^−1^·day^−1^) on whole skeletal muscle glycogen obtained from muscle biopsy samples at subgroups of 1.5–2.5, 2.5–3.5, 3.5–4.5 and > 5.5 g·kg^−1^·day^−1^ of CHO. Included study groups reported exercise status and CHO intake for at least 24 h pre‐muscle biopsy. Total effect estimates differ due to omission of subgroups with only 1 comparison.

Subgroups for Δ absolute CHO intake between conditions were 100–200, 200–300, 300–400, 400–500 and > 500 g, as the < 100 g subgroup only contained one pairwise comparison [16] (Experiment 2). There was a significant subgroup effect (*p* < 0.0001), as the magnitude of the significant effect of CHO intake on muscle glycogen increased in line with increases in Δ absolute CHO intake (Figure 6). The 200–300, 400–500 and > 500 g subgroups presented no significant heterogeneity (*I* ^2^ = 21%, *p* = 0.29; *I* ^2^ = 0%, *p* = 0.82; *I* ^2^ = 0%, *p* = 0.43, respectively), whilst the 100–200 g and 300–400 g groups also saw decreased heterogeneity compared to the overall effect estimate for crossover trials; however, heterogeneity was still considerable and significant (*I* ^2^ = 83%, *p* < 0.0001 and *I* ^2^ = 88%, *p* < 0.0001, respectively). Moreover, the number of studies contributing to each subgroup was low, particularly in those with low heterogeneity, as only 4, 3 and 2 pairwise comparisons contributed to the 200–300, 400–500 and > 500 g subgroups, respectively.

**FIGURE 6:**
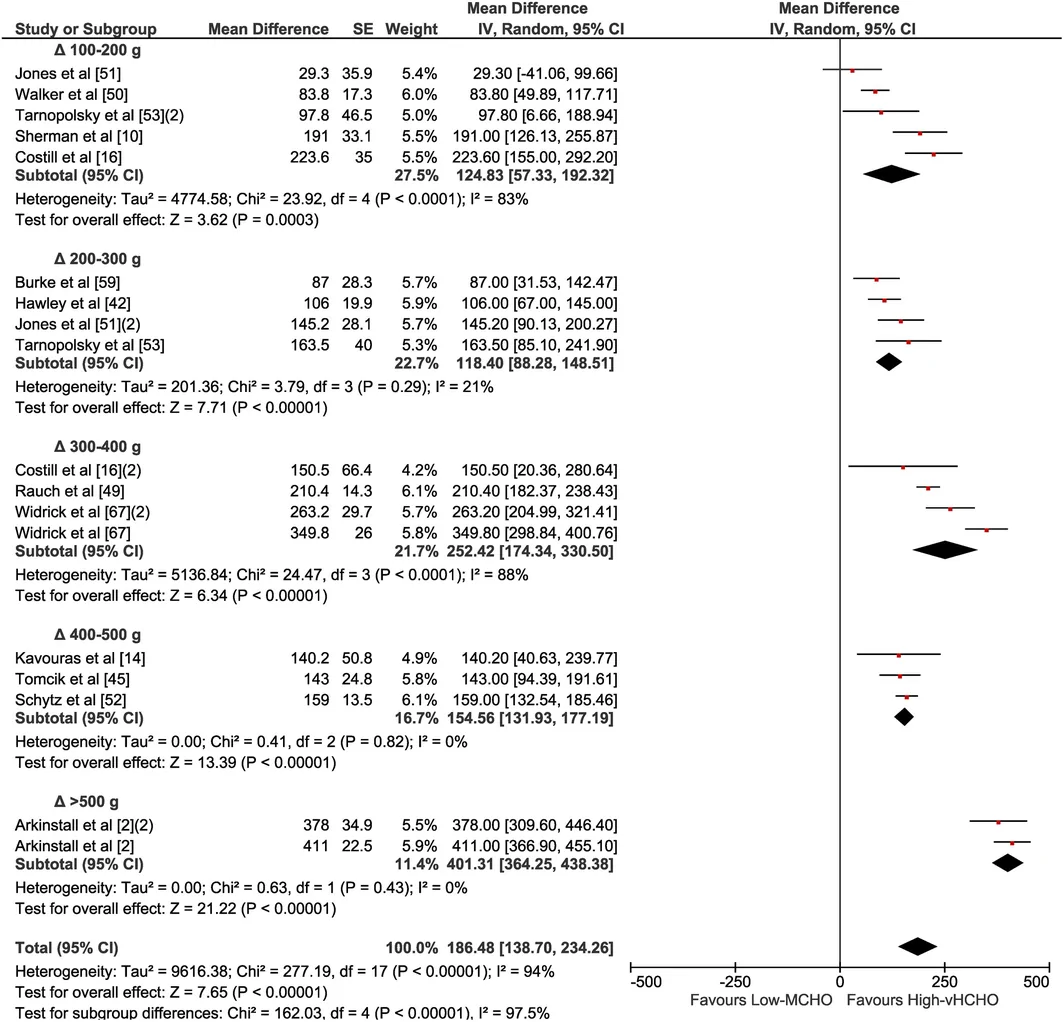
Forest plot of general inverse‐variance random‐effects meta‐analysis conducted in crossover design studies with subgroups for the Δ absolute CHO intake between defined low‐moderate and high‐very high CHO intake conditions (Low‐MCHO < 6.5 g·kg^−1^·day^−1^ vs. high‐vHCHO > 6.5 g·kg^−1^·day^−1^) on whole skeletal muscle glycogen obtained from muscle biopsy samples in 100 g·day^−1^ increments from 100 to > 500 g·day^−1^ of CHO. Included study groups reported exercise status and CHO intake for at least 24 h pre‐muscle biopsy. Total effect estimates differ due to omission of subgroups including only 1 comparison.

#### 3.3.2 CHO Loading Duration

There was a significant subgroup effect (*p* = 0.01) as the 24 and 48‐h loading periods had a greater impact on muscle glycogen compared to 72 and 96 h. The 48 and 72 h subgroups presented considerable and statistically significant heterogeneity (*p* < 0.0001 for both), whereas the 24 h and 96 h subgroups did not. However, effect estimates should be cautiously interpreted due to an uneven covariate distribution across loading durations and vastly different numbers of participants contributing to each subgroup (Table 2). Bussau et al. [9] reported no benefit in terms of glycogen content following a longer loading period (24 vs. 72 h), whereas others have shown a continued enhancement of muscle glycogen stores following high CHO intake over periods of 48 [25], 72 [34] and 96 h [33] versus 24 h.

**TABLE 2:** Summary statistics for subgroup analyses of loading duration and training status in the random effects meta‐analysis of randomized crossover design CHO loading studies, which reported exercise status and CHO intake for at least 24 h pre‐muscle biopsy.

Variable	Subgroups	Mean ΔCHO (g·kg^−1^·day^−1^)	*n* of comparisons (participants)	Mean difference (mmol·kg^−1^ DM)	Low 95% CI	High 95% CI	Overall effect	Heterogeneity *I* ^2^ (*p*)
Loading duration	24 h	3.1	2 (4)	207.7	147.0	268.4	*p* < 0.0001	0% (*p* = 0.33)
	48 h	6.0	7 (56)	255.0	160.7	349.2	*p* < 0.0001	97% (*p* < 0.0001)
	72 h	4.1	5 (38)	148.0	90.0	206.0	*p* < 0.0001	85% (*p* < 0.0001)
	96 h	3.4	3 (13)	106.4	59.0	153.7	*p* < 0.0001	40% (*p* = 0.19)
				Subgroup differences			***p* = 0.01**	
Training status	Trained	4.9	10 (47)	229.9	147.1	312.6	*p* < 0.0001	94% (*p* < 0.0001)
	Well trained	4.0	6 (42)	136.6	86.5	186.8	*p* < 0.0001	88% (*p* < 0.0001)
				Subgroup differences			*p* = 0.06	

*Note:* Subgroups with only one comparison were not pooled and are not shown. Subgroup analyses for Δ relative and Δ absolute CHO intake are presented in Figures 5 and 6. Sex and biopsy‐location subgroup analyses were not performed due to uneven covariate distributions. *p* values indicate significance between conditions with bold values the threshold for significance ≤ 0.01.

Abbreviations: CHO, carbohydrates; CI, confidence interval; h, hours; *n*, number of pairwise comparisons/participants in each subgroup; wk, week.

#### 3.3.3 Training Status

Subgroup analysis was attempted for participant training status; however, only trained and well‐trained subgroups contained more than one pairwise comparison. There was no significant subgroup difference (*p* = 0.06), and CHO intake between study groups in the trained versus well‐trained subgroup was similar (mean ΔCHO between comparisons of 4.9 vs. 4.0 g·kg^−1^·day^−1^, respectively). However, both subgroups presented significant heterogeneity (*p* < 0.0001 and *I* ^2^ ≥ 88%, for both; Table 2), and the covariate distribution was uneven, as an additional 24 participants contributed to the endurance trained subgroup. Individual studies that investigated differences in glycogen concentrations following 72 h of a very high CHO intake in trained versus untrained participants [13, 17] (Experiment 1) showed significantly greater glycogen stores in trained versus untrained individuals (583.3 ± 132.7 vs. 287.1 ± 37.8 and 792.1 ± 90.5 vs. 584.6 ± 37.4 mmol·kg^−1^ DM, respectively). Further, Greiwe et al. [18] reported significantly greater muscle glycogen from the same untrained participants following a repeated CHO loading protocol pre and post 10‐week endurance cycling training program (428.5 ± 85.7 and 795.6 ± 124.9 mmol·kg^−1^ DM, respectively).

#### 3.3.4 Exercise Pre‐ and During Loading

Kochan et al. [33] and Nelson et al. [76] compared depleting exercise versus resting conditions specifically using one‐legged models in untrained participants. Both studies reported significantly higher muscle glycogen concentrations in the exercised leg compared to the non‐exercised control. Whereas numerous others have reported similar increases in muscle glycogen concentration when a high or very high CHO intake (8.1–10.5 g·kg^−1^·day^−1^) was not preceded by specific glycogen depleting exercise [9, 30, 42, 49, 54, 63], however, these were trained individuals and likely completed habitual training in the 24–36 h pre‐intervention. Between study comparisons suggest exercise during the loading period does not negatively impact glycogen content, provided exercise is light‐to‐moderate intensity and relatively short duration (≤ 60 min), replicating a pre‐competition taper [53, 54, 68] and CHO intake is very high [51].

#### 3.3.5 Other Considered Covariates

No subgroup analysis was attempted for participant sex or muscle biopsy location due to substantially uneven covariate distributions between categorized subgroups (1 study recruited females only, 13 recruited males only and 4 recruited both; 2 studies biopsied the *gastrocnemius* whilst the remaining 16 took muscle samples from the *vastus lateralis*). When CHO intake was matched, males and females appear to have the same capacity to increase muscle glycogen stores [50, 53]. Impey et al. [31] reported lower glycogen concentrations in the *gastrocnemius* of female participants compared to males; however, no differences occurred in the *vastus lateralis*. When other covariates were similar (CHO intake, loading duration and participant training status), studies that collected biopsies from the *gastrocnemius* [10, 13, 16, 35, 72] have reported comparable glycogen content to those collecting samples from the *vastus lateralis* [3, 26, 65].

### 3.4 Risk of Bias Results

Of the 42 randomized experiments, 24 studies posed some concerns (Table 3), primarily due to lack of detail regarding randomization and allocation concealment (Domain 1), as methods were inadequately described. The remaining 18 randomized trials were deemed low risk. Of the 21 non‐randomized trials assessed using the ROBINS‐I (Table 4), 13 studies posed a serious risk of bias due to confounding variables (Domain 1) as participant training status, exercise status ≤ 24 h pre‐ and during the intervention or menstrual cycle phase were not controlled (or the control was inadequately described). The remaining eight trials posed the lowest overall risk of bias, except for concerns regarding uncontrolled confounding associated with the observational/non‐randomized nature of these studies.

**TABLE 3:** Cochrane risk of bias tool for randomized trials (RoB 2) included within systematic review descriptive synthesis.

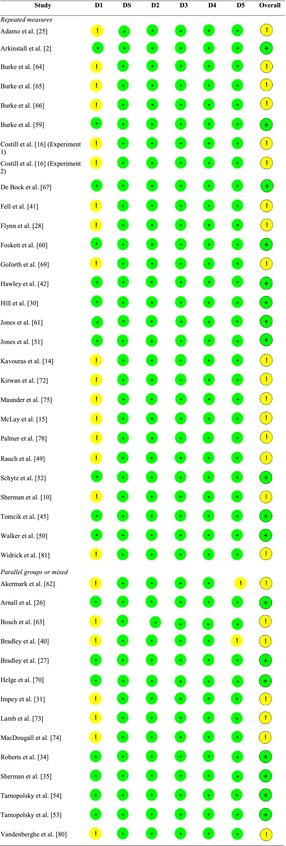

*Note:* Included studies reported exercise status and CHO intake for at least 24 h pre‐muscle biopsy. 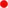, 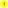, and 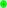 correspond to high risk, some concerns and low risk of bias, respectively.

Abbreviations: D1, randomization; D2, deviation from intended intervention; D3, missing data; D4, measurement of outcome; D5, selection of reported results; DS, period and carryover effects.

**TABLE 4:** Cochrane risk of bias tool for non‐randomized trials (ROBINS‐I) included within systematic review descriptive synthesis.

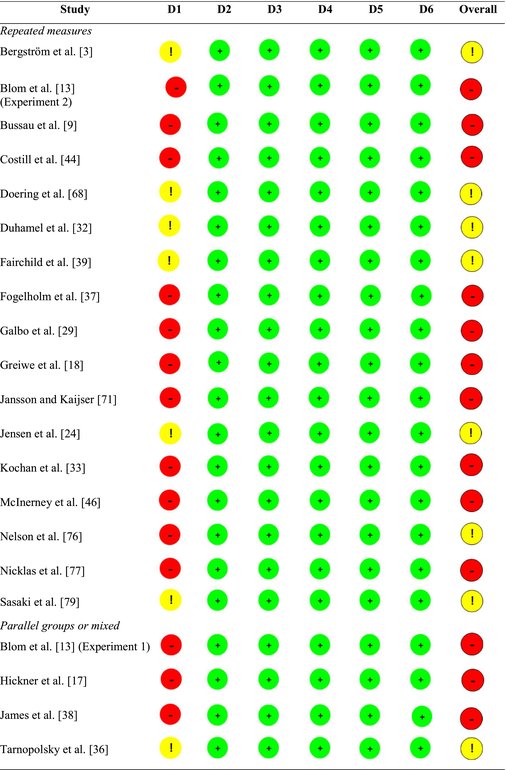

*Note:* Included studies reported exercise status and CHO intake for at least 24 h pre‐muscle biopsy. In line with the ROBINS‐I criteria 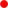, 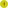, and 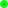 correspond to serious, moderate and low risk of bias. For D1 and overall risk of bias, 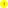 refers to low risk except for concerns about uncontrolled confounding associated with observational and non‐randomized study designs.

Abbreviations: D1, confounding; D2, classification of interventions; D3, selection of participants; D4, missing data; D5, measurement of outcome; D6, selection of reported results.

## 4 Discussion

Muscle glycogen is recognized as critical to optimize exercise capacity in most endurance sport disciplines and strategies to increase glycogen storage have been developed over half a century of research; however the relationship between dietary CHO intake and muscle glycogen concentration is not well understood. Therefore, this review and meta‐analysis aimed to describe and quantify the relationship between pre‐exercise dietary CHO intake and skeletal muscle glycogen, whilst identifying key covariates of glycogen concentration. Exploratory regression analyses indicated a linear dose–response relationship between dietary CHO intake and muscle glycogen concentration, with a hierarchy regarding covariates. Quantity of CHO ingested, training status, loading duration and exercise (pre‐ and during loading) were all effectors of muscle glycogen. Meta‐analyses revealed considerable and significant heterogeneity, which resulted from confounding of important covariates across studies. More research is required to truly determine whether an optimal CHO loading strategy for muscle glycogen supercompensation exists.

This systematic review and meta‐analysis, for the first time, suggests a significant linear dose–response relationship between dietary CHO intake and skeletal muscle glycogen, as relative CHO intake predicted 49% of the variation in muscle glycogen in endurance trained individuals (Figure 2b), which, in agreement with other reviews within this topic [8, 12, 43], suggests increased quantity of CHO ingested is one of the most important factors to achieve supercompensation of muscle glycogen stores. In line with this, subgroup analyses revealed Δ relative CHO intake subgroups increased muscle glycogen in a stepwise manner, with a possible saturation point, as muscle glycogen only marginally increased in the > 5.5 g·kg^−1^·day^−1^ subgroup (Figure 5). Considering habitual CHO intakes of trained individuals (~3–6 g·kg^−1^·day^−1^ dependent on training demands), a 5.5 g·kg^−1^·day^−1^ increase would achieve an intake of 8.5–11.5 g·kg^−1^·day^−1^ of CHO, which corresponds with contemporary CHO loading guidelines [11] and a previously suggested saturation point for muscle glycogen (7–10 g·kg^−1^·day^−1^) [8, 11, 65]. However, in contrast, subgroup analysis for Δ absolute CHO intake and regression analyses suggested higher CHO intakes are superior for maximization of glycogen stores pre‐competition, as the > 500 g·day^−1^ subgroup had the greatest enhancement of glycogen stores (+401.3 mmol·kg^−1^ DM), and both simple linear and second‐degree polynomial regression models consistently suggested a linear dose–response relationship (Figures 2 and 3), with no apparent saturation point or plateau. The linearity of the relationship weakened somewhat as CHO intake increased > 10 g·kg^−1^·day^−1^ (Figure 2e) suggesting the true saturation point may be higher than previously suggested [8], at least in endurance trained individuals. However, it should be considered only five study groups contributed to CHO intakes of ~12 g·kg^−1^·day^−1^; therefore, this slight plateau could also be due to lack of information at higher CHO intakes (Figures 2b and 3b).

The discrepancy between subgroup analyses of the Δ CHO consumed highlights the challenging nature of interpreting CHO loading research, particularly as both analyses display considerable and statistically significant heterogeneity across multiple subgroups, uneven covariate distributions and small numbers of study pairwise comparisons contributing to each subgroup (below the *n* = 10 recommended by statisticians; Figures 5 and 6; [19]). As such, the exact magnitude of the relationship between dietary CHO intake and muscle glycogen remains unclear. Conversely, exploratory regression analyses indicate a dose–response relationship, and it remains to be determined whether the previously suggested “ceiling” effect truly exists [65]. Ultimately, irrespective of how CHO intake was expressed (relative or absolute), a marked increase in CHO ingestion appears the most important factor to attain a significant enhancement or supercompensation of muscle glycogen stores (Figures 2 and 3).

Meta‐analysis results suggest 24–48 h was sufficient to achieve muscle glycogen supercompensation, whereas longer loading periods of 72 and 96 h provided no further benefit (Table 2). Bussau et al. [9] also claimed 24 h was adequate when CHO intake was very high (10.2 g·kg^−1^), as 72 h provided no further increase in muscle glycogen. However, the true loading period in the previous study was ~36 h (as the final habitual training session was ~12 h pre‐CHO loading), whilst the current review quantitative effect estimates are untrustworthy due to considerable and statistically significant heterogeneity, small numbers of studies contributing to each subgroup and confounding (mean ΔCHO dose differed across subgroups); therefore, glycogen concentration cannot be attributed to loading duration alone (Table 2), and an optimal loading duration cannot be determined from this analysis. Studies included within the descriptive synthesis showed a continued increase of glycogen stores with 48–72 versus 24 h when CHO intakes were > 8 g·kg^−1^·day^−1^ [10, 16, 25, 33, 34] (Experiment 1). Additionally, others have only reported replenishment of glycogen to expected baseline concentrations (~400 mmol·kg^−1^ DM) following 24 h of increased CHO intake post‐glycogen depletion [6, 33, 74], however, these participants were not endurance trained. Fairchild et al. [39] reported one of the highest glycogen values (862.2 ± 57.0 mmol·kg^−1^ DM) following 10.3 g·kg^−1^ of CHO for 24 h in endurance trained participants, suggesting when all factors are optimal (very high CHO intake, high glycaemic index CHO, heightened post‐exercise glycogen resynthesis, endurance trained participants), it is possible to achieve glycogen supercompensation within 24 h. Nevertheless, in most cases ~1.5‐fold enhancement of stores occurs within 24 h [64, 65, 66]. Therefore, 36–48 h of a high to very high CHO intake (> 8 g·kg^−1^·day^−1^) can be considered a practical recommendation to significantly increase pre‐competition glycogen stores.

It is well‐established that high glycaemic index CHO sources are superior for achieving a greater enhancement of glycogen synthesis and concentration during shorter recovery periods (< 24 h) [64, 82]. High glycaemic index CHO sources provide readily available glucose molecules for absorption within the gut, resulting in higher blood glucose and insulin responses, greater glucose uptake by the muscle, and increased glycogen synthesis compared to low glycaemic index CHO sources, which require longer to digest and absorb, and do not elicit the same blood glucose response [8, 64, 82]. Costill et al. [16] (Experiment 1) suggested there was no effect of consuming simple compared to complex CHO sources over a 24 h loading period; however, the classification of complex and simple CHO sources does not correspond to high and low glycaemic index, as complex CHO can still be high glycaemic index [83]. This likely contributed to the lack of differences between conditions reported in the previous study, and unfortunately foods consumed by participants were not adequately described to judge glycaemic index [8, 16] (Experiment 1). Based on existing evidence, athletes implementing shorter duration loading periods pre‐competition (< 36 h) should primarily consume high glycaemic index CHO sources and take advantage of the acute phase of increased post‐exercise resynthesis. For athletes using longer loading periods (> 36 h), a mixed healthy diet with a combination of low and high glycaemic index foods would likely be adequate, as longer recovery periods appear to minimize the importance of CHO type [8, 84]. However, as achieving very high CHO intakes can be challenging due to potential gastrointestinal discomfort [53, 73], athletes should supplement nutritional intake with low fiber, high glycaemic index CHO sources to support tolerability.

Training status did not significantly impact glycogen concentration; however, subgroup effect estimates are untrustworthy due to high subgroup heterogeneity and unbalanced covariate distributions (Table 2). A previous meta‐analysis of CHO intake and muscle glycogen reported that under periods of normal and high CHO availability for every 10 mL·kg^−1^·min^−1^ increase in V̇O_2max_, individuals have 67 ± 15 and 123 ± 42 mmol·kg^−1^ DM greater glycogen concentrations at rest [43]. In agreement, previous studies have consistently shown endurance trained individuals have a greater ability to store muscle glycogen post‐exercise with increased dietary CHO intake [13, 17, 18], due to higher glycogen synthesis rates post‐exercise, primarily due to greater GLUT4 and glycogen synthase activity [13, 17, 18]. Furthermore, endurance trained individuals have a greater composition of type I muscle fibers, and considering glycogen is preferentially stored in the intermyofibrillar space of type I fibers following a high CHO intake for 72 h [85], theoretically trained individuals have greater capacity for glycogen storage. Interestingly, regression data showed no significant linear relationship between CHO intake and muscle glycogen in well‐trained participants (Figures 2c and 3c). One potential reason behind this could be as training status increases, required CHO intake to achieve maximization of glycogen stores diminishes due to upregulation of post‐exercise CHO storage via greater glycogen synthase and GLUT4 activity post‐exercise. However, results were likely affected by the smaller number of study groups in the well‐trained regression analysis, particularly at low and very high CHO intakes, where no studies fed < 4.7 or > 12.0 g·kg^−1^·day^−1^ of CHO (Figure 2c), limiting the ability to detect potential linearity through a smaller spread across CHO intakes compared to other regression models.

Conducting exercise pre‐CHO loading, either as a specific glycogen depleting session or a high‐intensity exercise stimulus increases muscle glycogen synthesis rates compared to rested conditions as glycogen utilization drives localized glycogen resynthesis in the exercised muscle through increased glycogen synthase activity [6, 33, 76, 86]. However, it should be noted that previous studies were conducted in relatively untrained participants, as others have shown a very high CHO intake (9–10 g·kg^−1^·day^−1^) without challenging exercise pre‐CHO loading to increase glycogen concentrations in endurance trained individuals [9, 30, 49]. However, considering the beneficial mechanisms of an exercise stimulus for glycogen synthesis, athletes should strategically implement CHO loading around pre‐competition training schedules, where elite athletes typically complete their final high‐intensity training sessions 2–3 days prior (unpublished observations from the field), which would provide a strong stimulus to drive glycogen resynthesis. In combination with an aggressive acute post‐exercise refeed of CHO (1–1.2 g·kg^−1^·h^−1^ for the first 4 h [11]) and a high‐very high CHO intake for the subsequent 36–48 h, in theory, this would optimize glycogen synthesis and maximize pre‐competition glycogen stores (provided no further moderate‐to‐high‐intensity exercise was conducted).

Exercise during the loading period itself also requires consideration, as training at a moderate‐to‐high intensity in the days before competition would utilize glycogen stores, potentially leading to suboptimal glycogen concentrations for endurance performance. Between study comparisons suggest, provided CHO intake is very high (e.g., 10 g·kg^−1^) and training intensity is low‐to‐moderate (e.g., pre‐competition taper), there is no negative effect of training during the loading period on muscle glycogen concentration [10, 51, 53, 68], which is common practice for endurance athletes to maintain training adaptations [87]. Interestingly, 65% of studies rested participants during periods of increased CHO intake, which does not represent the real‐world pre‐competition practices of athletes. Furthermore, those who included light‐to‐moderate‐intensity exercise sessions as a simulated pre‐competition taper, collected no measurements during exercise to describe physiological or metabolic responses [10, 53, 68]. Considering increased glycogen stores augment the contribution of glycogen to CHO oxidation and exercise energy expenditure at the same relative exercise intensity [1, 2], glycogen utilization during exercise conducted during the loading period would be increased (provided intensity and duration were consistent). Alternatively, these sessions also provide an exercise stimulus, with augmented post‐exercise synthesis rates providing an opportunity to replenish glycogen stores (provided post‐exercise CHO ingestion was adequately increased and other effectors [timing and type] were optimal), which could explain the high glycogen concentrations reported in the previous studies [10, 51, 53, 68]. Nevertheless, these suggestions are speculative and require further investigation.

Tarnopolsky et al. [54] suggested participant biological sex was a covariate of muscle glycogen, as females were shown to have an impaired ability to store muscle glycogen. However, differences were likely due to variable CHO intakes between conditions as opposed to biological sex (6.4 vs. 8.2 g·kg^−1^·day^−1^ CHO for females and males, respectively). When relative or absolute CHO intake (g·kg^−1^·day^−1^ or g·day^−1^, respectively) was similar between males and females, either within the same study [31, 38, 53] or between trials [15, 50, 77], females had the same capacity for muscle glycogen storage (Table 1), suggesting CHO guidelines are applicable to both sexes. However, effects of female menstrual cycle phase on muscle glycogen storage are inconclusive, as some reports suggest greater glycogen stores for females in the mid‐luteal compared to mid‐follicular phase [15, 77]. However, when CHO intake was 8–10 g·kg^−1^·day^−1^, no differences were apparent between menstrual cycle phases [15, 38]. These mixed results justify further investigation; however, the practical relevance is debatable due to alignment of competition days and menstrual cycle phase being beyond the control of female athletes.

A limitation of the quantitative analysis is the observational nature of subgroup analyses [19]; however, caution has been suggested throughout regarding interpretation of effect estimates, with transparency in terms of covariate distribution (number of pairwise comparisons and participants) and heterogeneity. It could be argued meta‐regression may have been more statistically effective in assessing effects of covariates; however, due to the confounded nature of covariates that contributed to heterogeneity in muscle glycogen (CHO intake, loading duration, and training status were not classifiable due to inconsistency across the different levels of these factors), a meta‐regression was deemed inappropriate. Another limitation of the current systematic review and meta‐analysis is that beyond heterogeneity, no other quality of evidence criteria were assessed (e.g., indirectness, imprecision or publication bias).

This systematic review and meta‐analysis highlights a linear dose–response relationship between dietary CHO intake and muscle glycogen, with the saturation point remaining unclear due to a lack of information at higher CHO intakes (≥ 12 g·kg^−1^·day^−1^). A multifaceted heterogeneity exists, as the quantity of CHO ingested (expressed as g·kg^−1^·day^−1^ or g·day^−1^), loading duration, training status, and exercise (pre‐ and during the loading period) were all effectors of glycogen concentration. Therefore, achieving an optimization of pre‐competition glycogen stores is a balancing act of what is optimal for glycogen synthesis and what is practical for athletes to implement in the real world, which is achievable with different combinations of these key effectors. Findings reveal a hierarchy, as dietary CHO intake (> 8 g·kg^−1^·day^−1^ for 36–48 h) was the most important factor to significantly increase muscle glycogen stores. Despite specific depleting exercise pre‐loading not being a pre‐requisite for endurance trained individuals, athletes should strategically implement loading strategies around their training sessions pre‐competition to efficiently maximize glycogen stores. However, significant heterogeneity in past research introduces uncertainty in these recommendations, and further well‐controlled studies are required to determine the true optimal pre‐exercise CHO intake to maximize glycogen concentrations, and what effect this has on endurance exercise performance.

## 5 Perspectives

Future research should aim to discover the upper limit of CHO ingestion during loading with intakes ≥ 12 g·kg^−1^·day^−1^ to identify the saturation point for both muscle glycogen and gastrointestinal tolerance in endurance trained individuals. The descriptive element of this systematic review collates the characteristics of existing CHO loading research, which identifies key trends and potential avenues for new research within this topic. Examples include the limited replication of athlete pre‐competition training practices and poor representation of females and master athletes (no study groups aged > 37 years old). As such, the effects of menstrual cycle and age on the ability to achieve glycogen supercompensation remain unclear. It should be noted that this review and meta‐analysis focused on “optimizing” CHO loading in the context of maximizing muscle glycogen stores; however, in many sports (e.g., team sports) and some endurance activities (e.g., race‐walking < 20 km), this is unnecessary, as adequate fuelling through the normalization of muscle glycogen stores can be achieved with intakes from 6 to 10 g·kg^−1^·day^−1^.

## Funding

The authors have nothing to report.

## Conflicts of Interest

The authors declare no conflicts of interest.

## Supporting information


**Table S1:** Sensitivity analysis to assess goodness of fit of nonlinear regression models used to describe the relationship between dietary CHO intake and muscle glycogen using studies included within systematic review descriptive synthesis.
**Table S2:** Linear and nonlinear sensitivity analysis to describe the relationship between dietary CHO intake and muscle glycogen, using only independent observations.
**Table S3:** Sensitivity analysis summary statistics for multiple vs. combined pairwise comparisons within meta‐analysis which quantified the relationship between dietary CHO intake and muscle glycogen. Included studies reported exercise status and CHO intake for at least 24 h pre‐muscle biopsy.
**Table S4:** Sensitivity analysis summary statistics for fixed versus random effects meta‐analytic models using imputations with different borrowed correlation coefficients. Meta‐analysis quantified the relationship between dietary CHO intake and muscle glycogen using studies which reported exercise status and CHO intake for at least 24 h pre‐muscle biopsy.

## Data Availability

The data that support the findings of this study are available from the corresponding author upon reasonable request.
