# Supplementary material for: Dietary Carbohydrate Intake and Skeletal Muscle Glycogen: A Systematic Review and Meta‐Analysis to Determine Optimal Carbohydrate Loading Strategies
Source: Scand J Med Sci Sports. 2026 Oct 3;36(10):e70379. doi: 10.1111/sms.70379 (PMC13633599; doi:10.1111/sms.70379)
Supplement: Supplementary file 1 [file SMS-36-e70379-s001.docx]

**SUPPORTING INFORMATION – Jones et al. Dietary carbohydrate intake and skeletal muscle glycogen: A systematic review and meta-analysis to determine optimal carbohydrate loading strategies**

**Keyword algorithm generated by each database within the search strategy**

*PubMed****:***

("CHO"[All Fields] OR ("carbohydrate s"[All Fields] OR "carbohydrated"[All Fields] OR "carbohydrates"[Supplementary Concept] OR "carbohydrates"[All Fields] OR "carbohydrate"[All Fields] OR "carbohydrates"[MeSH Terms]) OR ("maltodextrin"[Supplementary Concept] OR "maltodextrin"[All Fields] OR "maltodextrine"[All Fields] OR "maltodextrins"[All Fields]) OR ("glucose"[Supplementary Concept] OR "glucose"[All Fields] OR "glucose"[MeSH Terms] OR "glucoses"[All Fields] OR "glucose s"[All Fields]) OR ("sucrose"[Supplementary Concept] OR "sucrose"[All Fields] OR "sucrose"[MeSH Terms] OR "sucroses"[All Fields]) OR ("fructose"[Supplementary Concept] OR "fructose"[All Fields] OR "fructose"[MeSH Terms] OR "fructoses"[All Fields])) AND ("load*"[All Fields] OR "supercompensat*"[All Fields]) AND ("muscle s"[All Fields] OR "muscles"[MeSH Terms] OR "muscles"[All Fields] OR "muscle"[All Fields] OR ("glycogen"[Supplementary Concept] OR "glycogen"[All Fields] OR "glycogen"[MeSH Terms] OR "glycogenic"[All Fields] OR "glycogens"[All Fields]) OR ("store"[All Fields] OR "store s"[All Fields] OR "stored"[All Fields] OR "stores"[All Fields] OR "storing"[All Fields]) OR ("storage"[All Fields] OR "storages"[All Fields])) AND ("concentrate"[All Fields] OR "concentrated"[All Fields] OR "concentrates"[All Fields] OR "concentrating"[All Fields] OR "concentration"[All Fields] OR "concentrations"[All Fields] OR ("content"[All Fields] OR "contents"[All Fields]) OR ("metabolism"[MeSH Terms] OR "metabolism"[All Fields] OR "synthesis"[All Fields]) OR "resynthesis"[All Fields] OR ("utilisation"[All Fields] OR "statistics and numerical data"[MeSH Subheading] OR ("statistics"[All Fields] AND "numerical"[All Fields] AND "data"[All Fields]) OR "statistics and numerical data"[All Fields] OR "utilization"[All Fields] OR "utilisations"[All Fields] OR "utilise"[All Fields] OR "utilised"[All Fields] OR "utilises"[All Fields] OR "utilising"[All Fields] OR "utilities"[All Fields] OR "utility"[All Fields] OR "utilizations"[All Fields] OR "utilize"[All Fields] OR "utilized"[All Fields] OR "utilizer"[All Fields] OR "utilizers"[All Fields] OR "utilizes"[All Fields] OR "utilizing"[All Fields]) OR ("accumulate"[All Fields] OR "accumulated"[All Fields] OR "accumulates"[All Fields] OR "accumulating"[All Fields] OR "accumulation"[All Fields] OR "accumulations"[All Fields] OR "accumulative"[All Fields] OR "accumulator"[All Fields] OR "accumulators"[All Fields]))

*Cochrane:* Title, Abstract and Keywords applied, with search word variations selected.

(CHO OR carbohydrate OR maltodextrin OR glucose OR sucrose OR fructose) AND (Load* OR supercompensat*) AND (muscle OR glycogen OR stores OR storage) AND (concentration OR content OR synthesis OR resynthesis OR utilization OR accumulation).

*Web of Science:*

(((((((((TS = CHO) OR (TS = carbohydrate)) OR (TS = maltodextrin)) OR (TS = glucose)) OR (TS = sucrose)) OR (TS = fructose)) AND ((TS = Load*) OR (TS = supercompensat*))) AND ((((TS = muscle) OR (TS = glycogen)) OR (TS = stores)) OR (TS = storage))) AND ((((((TS = concentration) OR (TS = content)) OR (TS = synthesis)) OR (TS = resynthesis)) OR (TS = utilization)) OR (TS = accumulation)))

*SportsDiscus:* All fields selected for each line.

(CHO OR carbohydrate OR maltodextrin OR glucose OR sucrose OR fructose) AND

(Load* OR supercompensat*) AND

(muscle OR glycogen OR stores OR storage) AND

(concentration OR content OR synthesis OR resynthesis OR utilization OR accumulation).

*Scopus:* TITLE-ABS-KEY ((CHO OR carbohydrate OR maltodextrin OR glucose OR sucrose OR fructose) AND

(Load* OR supercompensat*) AND

(muscle OR glycogen OR stores OR storage) AND

(concentration OR content OR synthesis OR resynthesis OR utilization OR accumulation))

**Additional descriptive data**

*Muscle biopsy information.* Fifty-five trials collected muscle biopsy samples from the *vastus lateralis*, whilst 7 out of 63 took samples from the *gastrocnemius*, all of which were running models [1-5] (Blom et al. experiment 1 and 2). Impey et al. [6] was the only study to collect samples from both locations. Twelve studies took pre-CHO loading muscle biopsies to determine baseline glycogen concentrations [1, 7-17] (Blom et al. experiment 1). A total of 6, 13, 18 and 7 studies took muscle biopsies following 24, 48, 72 and 96 h of CHO loading, with others taking muscle biopsies following less common loading durations of 36 h [18-20], 84 h [13] or >96 h [16, 21]. Twelve studies collected muscle biopsies across multiple consecutive loading days [1-5, 8, 12, 22-25] (Blom et al. experiment 1 and 2, Costill et al. experiment 1).

*Baseline muscle glycogen concentrations at rest.* Thirteen trials reported a habitual or “baseline” muscle glycogen concentration (according to author description), which ranged from 328.9 to 518.0 mmol⋅kg^-1^ DM [1, 7-17, 26, 27] (Blom et al. experiment 1). Four of which did not control diet or exercise pre-biopsy [7, 10, 12, 14], 3 trials recorded habitual nutritional intake and allowed participants to continue training habitually [8, 9, 11], whereas others strictly controlled nutritional intake[1, 13, 15-17, 27](Blom et al. experiment 1).

**Regression models sensitivity analysis**

*Study design*. To determine whether inclusion of all study designs within the same regression models effected results, regression models were run for relative CHO intake and muscle glycogen with the inclusion of only repeated measures designs compared with results when all study designs were included. Repeated measures simple linear regression for recreationally active (F_1,23_ = 14.30, *r^2^* = 0.384, *P* = 0.001), endurance trained (F_1,71_ = 65.75, *r^2^* = 0.481, *P* < 0.001) and well trained (F_1, 24_ = 1.67, *r^2^* = 0.065, *P* = 0.208) were similar to results reported with inclusion of all study designs (F_1,42_ = 20.91, *r^2^* = 0.332, *P* < 0.001; F_1,75_ = 72.14, *r^2^* = 0.490, *P* < 0.001; and F_1, 24_ = 2.24, *r^2^* = 0.086, *P* = 0.147, respectively). Indicating results were robust to the inclusion of different study designs within the analysis. This was deemed more appropriate than excluding information from studies that utilised between subjects or mixed designs, as all other inclusion criteria were met and there was no other rationale for exclusion (e.g. measurement error).

*Polynomial regression models.* To determine the most appropriate nonlinear regression model, additional analyses were conducted with third degree polynomial models for relative and absolute CHO intake. Assessment of the Akaike Information Criterion (AIC) and adjusted *r^2^* indicated 2^nd^ degree polynomial models best fitted the pooled data (Table S1). Third degree polynomial models were unstable for absolute CHO intake, which was indicated by error messages in GraphPad Prism (version 10, Massachusetts, USA).

*Effect of violated statistical assumption.* The inclusion of multiple data points from the same study violates a statistical assumption of regression analyses (independence of observations and residuals). As such, to confirm the primary analysis was robust, additional sensitivity analyses were conducted to determine how the inclusion of data points exclusively from independent participant cohorts effected results. For repeated measures studies, only the final timepoint of each condition was included (i.e. a CHO loading condition with muscle biopsies following 24 and 72 h, only the 72 h timepoint was included). Thereafter, to limit risk of selection bias, in repeated measures design studies containing multiple conditions, the selected observation was randomly chosen for each study using an online random number generator (Table S2). As regression results were consistent across the sensitivity and primary analysis, results were deemed robust and the primary analysis was retained.

| **Table S1.** Sensitivity analysis to assess goodness of fit of nonlinear regression models used to describe the relationship between dietary CHO intake and muscle glycogen using studies included within systematic review descriptive synthesis. | | | | | | |
| --- | --- | --- | --- | --- | --- | --- |
| Polynomial Model | CHO intake | Training status | df | *r^2^* | Adjusted *r^2^* | AIC |
| 2^nd^ degree | Relative | Rec active | 41 | 0.332 | 0.300 | 434.1 |
| 3^rd^ degree |  |  | 40 | 0.339 | 0.290 | 436.2 |
|  |  |  |  |  |  |  |
| 2^nd^ degree | Relative | Endurance trained | 74 | 0.515 | 0.502 | 753.7 |
| 3^rd^ degree |  |  | 73 | 0.515 | 0.495 | 756.0 |
|  |  |  |  |  |  |  |
| 2^nd^ degree | Relative | Well trained | 23 | 0.092 | 0.013 | 264.4 |
| 3^rd^ degree |  |  | 22 | 0.115 | -0.005 | 266.8 |
|  |  |  |  |  |  |  |
| 2^nd^ degree | Absolute | Rec active | 39 | 0.297 | 0.260 | 418.2 |
| 3^rd^ degree |  |  | 38 | 0.309 | 0.254 | 420.0 |
|  |  |  |  |  |  |  |
| 2^nd^ degree | Absolute | Endurance trained | 74 | 0.497 | 0.483 | 756.6 |
| 3^rd^ degree |  |  | 73 | 0.500 | 0.479 | 758.4 |
|  |  |  |  |  |  |  |
| 2^nd^ degree | Absolute | Well trained | 22 | 0.047 | -0.039 | 254.2 |
| 3^rd^ degree |  |  | 21 | 0.051 | -0.084 | 257.2 |

| **Table S2.** Linear and nonlinear sensitivity analysis to describe the relationship between dietary CHO intake and muscle glycogen, using only independent observations. | | | | | | |
| --- | --- | --- | --- | --- | --- | --- |
| Regression model | CHO intake | Training status | df | F | *r^2^* | Significance |
| Linear | Relative | Rec active | 21 | 13.72 | 0.395 | ***P* = 0.001** |
|  |  | Endurance trained | 26 | 33.96 | 0.566 | ***P* < 0.001** |
| 2^nd^ degree polynomial | Relative | Rec active | 20 | 6.54 | 0.395 | ***P* = 0.007** |
|  |  | Endurance trained | 25 | 19.16 | 0.605 | ***P* < 0.001** |
| Linear | Absolute | Rec active | 19 | 16.14 | 0.347 | ***P* < 0.001** |
|  |  | Endurance trained | 26 | 31.85 | 0.551 | ***P* = 0.005** |
| 2^nd^ degree polynomial | Absolute | Rec active | 18 | 4.87 | 0.351 | ***P* = 0.020** |
|  |  | Endurance trained | 25 | 18.23 | 0.593 | ***P* < 0.001** |
| Degrees of freedom differ between relative and absolute CHO for recreationally active participants, as absolute CHO intake was not calculable for two conditions, which were excluded. | | | | | | |

***Meta-analysis imputations***

To quantify effect measures, mean difference and standard error of the mean difference was calculated using the equations below [28]:

1) $MD=M_{E}-M_{C}$
2) $SE (MD)=\frac{{SD}_{diff}}{\surd n}$

Where $MD$ is mean difference, $M_{E}$ is the mean of experimental group and $M_{C}$ is the mean of control group. *SE* is the standard error of the mean difference; ${SD}_{diff}$is the standard deviation of the differences and *n* is the number of participants. For parallel group trials, this was done using the calculator function in RevMan where between subject group means, *n* of participants and standard deviations were entered, and effect estimates were provided.

For crossover trials dependency (use of the same participants across experimental conditions) was accounted for to minimise unit of analysis error. Only two studies included within the analysis reported individual participant data [29, 30]. Authors from the 11 remaining crossover design experiments were contacted, and individual participant data was requested. Two groups of authors provided individual participant data [31, 32], others stated that individual data was not available, or a response was not received. As the required statistics are rarely reported in published articles, imputations were required using the following equations based on the information available in published reports [28]:

$${SD}_{diff}=\sqrt{{SD}_{E}^{2} +{SD}_{C}^{2}-(2 \times Corr \times{SD}_{E} \times{SD}_{C})}$$

Where ${SD}_{E}$ and ${SD}_{C}$ are the standard deviations of experimental and control group, respectively. *Corr* refers to a correlation coefficient which was borrowed from the studies which provided adequate statistical information [29-32]. Correlation coefficient was calculated as shown below:

$$Corr= \frac{{SD}_{E}^{2}+ {SD}_{C}^{2}-{SD}_{diff}^{2}}{2 \times{SD}_{E} \times{SD}_{C}}$$

A pooled borrowed correlation coefficient, taken from studies that provided individual participant information [29-32] was used for any studies where the necessary data was not available [2, 4, 26, 33-35].

***Combining groups***

Jones et al. [17] included three conditions (moderate, high and very high CHO), as such the high and very high CHO groups were combined using individual participant data, to generate one pairwise comparison. This was deemed more appropriate, as opposed to including the same measured group mean on multiple occasions (i.e. moderate vs high, and moderate vs very high) which would give rise to unit of analysis error [36]. Similarly, groups within mixed design studies that used CHO loading interventions within the same defined category of CHO intake (i.e. low-moderate or high-very high) were combined to create a single pairwise comparison which was included within the meta-analysis. Groups were combined using the following equations:

$$M_{combined}= \frac{n_{1}M_{1}+n_{2}M_{2}}{n_{1}+ n_{2}}$$

$${SD}_{combined}=\sqrt{\frac{\left( n_{1}-1 \right) {SD}_{1}^{2}+{\left( n_{2}-1 \right)SD}_{2}^{2}+ \frac{n_{1}n_{2}}{n_{1}{+ n}_{2}}(M_{1}^{2}+M_{2}^{2}-2M_{1}M_{2})}{n_{1}+ n_{2}-1}}$$

Where *n*, *M*, and *SD* are number of participants, mean and standard deviation, respectively. Experimental conditions of Tarnopolsky et al. [37] compared males versus females across two conditions where CHO intake for male and female conditions were defined as high and moderate, respectively. As such conditions within the same sex were combined (as above) to allow a single pairwise comparison of a moderate versus high CHO intake, which was a parallel groups comparison and was treated as such within the meta-analysis. Tarnopolsky et al. [38] compared males and females across 3 separate conditions of varied CHO intake. For males, conditions were defined as moderate, high and very high, with the high and very high conditions combined. For females conditions were moderate, moderate and high, with both moderate conditions combined. These groups were treated as independent crossover trials for the meta-analysis with dependency accounted for as previously described.

***Sensitivity analysis: Combined groups vs multiple pairwise comparisons***

Crossover trials containing four experimental conditions within the same study were presented within meta-analyses as separate pairwise comparisons [2, 33, 39]. This was deemed appropriate as these measures were following separate study conditions and the data used across multiple comparisons differed. Despite experimental conditions being different, this may give rise to unit of analysis error, as participants are the same individuals measured across multiple conditions, therefore study results are correlated. As such sensitivity analyses were conducted to determine whether combining groups to a single comparison per study (as recommended [36] and previously described) would have been more appropriate (Table S3). Analysis indicated no significant impact on overall effect or heterogeneity.

| **Table S3.** Sensitivity analysis summary statistics for multiple vs combined pairwise comparisons within meta-analysis which quantified the relationship between dietary CHO intake and muscle glycogen. Included studies reported exercise status and CHO intake for at least 24 h pre-muscle biopsy. | | | | | | |
| --- | --- | --- | --- | --- | --- | --- |
| Study design | Pairwise comparisons  (n) | Mean diff  (mmol⋅kg^-1^ DM) | Low 95% CI | High 95% CI | Overall effect | I^2^ |
| Within groups |  |  |  |  |  |  |
|  | Multiple  (17) | 191.9 | 142.8 | 241.1 | *P* < 0.0001 | 94% |
|  | Combined  (14) | 168.8 | 123.4 | 214.1 | *P* < 0.0001 | 92% |

**Sensitivity analysis: Fixed vs Random effects models and imputed correlation coefficients**

A sensitivity analysis was performed to ensure decisions regarding the type of model (fixed versus random effects) and the chosen correlation coefficient from imputations had minimal impact on overall meta-analysis results. This was done by running the analysis on two more occasions using lower and higher imputed correlation coefficients of 0.5499 and 0.9460 borrowed from data presented by Schytz et al. [32] and Rauch et al. [29], respectively. The results of this analysis are presented in Table S4.

| **Table S4.** Sensitivity analysis summary statistics for fixed versus random effects meta-analytic models using imputations with different borrowed correlation coefficients. Meta-analysis quantified the relationship between dietary CHO intake and muscle glycogen using studies which reported exercise status and CHO intake for at least 24 h pre-muscle biopsy. | | | | | | | | |
| --- | --- | --- | --- | --- | --- | --- | --- | --- |
| Model | Study design | Correlation coefficient | n | Mean diff  (mmol⋅kg^-1^ DM) | Low 95% CI | High 95% CI | Overall effect | I^2^ |
| Fixed effects |  |  |  |  |  |  |  |  |
|  | Within groups | 0.55 | 111 | 177.7 | 165.0 | 190.4 | *P* < 0.001 | 91% |
|  |  | 0.81 | 111 | 184.7 | 173.3 | 196.1 | *P* < 0.001 | 94% |
|  |  | 0.95 | 111 | 181.3 | 171.8 | 190.8 | *P* < 0.001 | 96% |
|  | Parallel groups | N/A | 81 | 85.7 | 56.8 | 114.6 | *P* < 0.001 | 92% |
|  |  |  |  |  |  |  |  |  |
| Random effects |  |  |  |  |  |  |  |  |
|  | Within group | 0.55 | 111 | 191.3 | 144.9 | 237.7 | *P* < 0.001 | 91% |
|  |  | 0.81 | 111 | 191.9 | 142.8 | 241.1 | *P* < 0.001 | 94% |
|  |  | 0.95 | 111 | 192.2 | 141.9 | 242.4 | *P* < 0.001 | 96% |
|  | Parallel groups | N/A | 81 | 107.6 | -10.5 | 225.7 | *P* = 0.07 | 92% |
|  |  |  |  |  |  |  |  |  |
|  | n, number of participants; CI, confidence interval; SE, standard error. | | | | | | | |

**References**

1.	Blom PC, Costill DL, Vollestad NK. Exhaustive running: inappropriate as a stimulus of muscle glycogen super-compensation. Med Sci Sports Exerc. 1987 Aug;19(4):398-403.

2.	Costill DL, Sherman WM, Fink WJ, Maresh C, Witten M, Miller JM. The role of dietary carbohydrates in muscle glycogen resynthesis after strenuous running. Am J Clin Nutr. 1981 Sep;34(9):1831-6.

3.	Kirwan JP, Costill DL, Mitchell JB, Houmard JA, Flynn MG, Fink WJ, et al. Carbohydrate balance in competitive runners during successive days of intense training. J Appl Physiol (1985). 1988 Dec;65(6):2601-6.

4.	Sherman WM, Costill DL, Fink WJ, Miller JM. Effect of exercise-diet manipulation on muscle glycogen and its subsequent utilization during performance. Int J Sports Med. 1981 May;2(2):114-8.

5.	Sherman WM, Costill DL, Fink WJ, Hagerman FC, Armstrong LE, Murray TF. Effect of a 42.2-km footrace and subsequent rest or exercise on muscle glycogen and enzymes. J Appl Physiol Respir Environ Exerc Physiol. 1983 Oct;55(4):1219-24.

6.	Impey SG, Jevons E, Mees G, Cocks M, Strauss J, Chester N, et al. Glycogen Utilization during Running: Intensity, Sex, and Muscle-Specific Responses. Med Sci Sports Exerc. 2020 Sep;52(9):1966-75.

7.	Arnall DA, Nelson AG, Quigley J, Lex S, Dehart T, Fortune P. Supercompensated glycogen loads persist 5 days in resting trained cyclists. Eur J Appl Physiol. 2007 Feb;99(3):251-6.

8.	Bussau VA, Fairchild TJ, Rao A, Steele P, Fournier PA. Carbohydrate loading in human muscle: an improved 1 day protocol. Eur J Appl Physiol. 2002 Jul;87(3):290-5.

9.	Fairchild TJ, Fletcher S, Steele P, Goodman C, Dawson B, Fournier PA. Rapid carbohydrate loading after a short bout of near maximal-intensity exercise. Med Sci Sports Exerc. 2002 Jun;34(6):980-6.

10.	Goforth HW, Jr., Arnall DA, Bennett BL, Law PG. Persistence of supercompensated muscle glycogen in trained subjects after carbohydrate loading. J Appl Physiol (1985). 1997 Jan;82(1):342-7.

11.	James AP, Lorraine M, Cullen D, Goodman C, Dawson B, Palmer TN, et al. Muscle glycogen supercompensation: absence of a gender-related difference. Eur J Appl Physiol. 2001 Oct;85(6):533-8.

12.	Kochan RG, Lamb DR, Lutz SA, Perrill CV, Reimann EM, Schlender KK. Glycogen synthase activation in human skeletal muscle: effects of diet and exercise. Am J Physiol. 1979 Jun;236(6):E660-6.

13.	Lamb DR, Snyder AC, Baur TS. Muscle glycogen loading with a liquid carbohydrate supplement. Int J Sport Nutr. 1991 Mar;1(1):52-60.

14.	Nelson AG, Arnall DA, Kokkonen J, Day R, Evans J. Muscle glycogen supercompensation is enhanced by prior creatine supplementation. Med Sci Sports Exerc. 2001 Jul;33(7):1096-100.

15.	Fogelholm GM, Tikkanen HO, Naveri HK, Naveri LS, Harkonen MH. Carbohydrate loading in practice: high muscle glycogen concentration is not certain. Br J Sports Med. 1991 Mar;25(1):41-4.

16.	Helge JW, Watt PW, Richter EA, Rennie MJ, Kiens B. Partial restoration of dietary fat induced metabolic adaptations to training by 7 days of carbohydrate diet. J Appl Physiol (1985). 2002 Nov;93(5):1797-805.

17.	Jones RO, Areta JL, Bennett S, Pugh J, Louis JB. Dose-Response of Dietary Carbohydrate Intake on Skeletal Muscle Glycogen, Gastrointestinal Comfort and Body Composition in Endurance-Trained Individuals in Simulated Preparation for Competition. Scand J Med Sci Sports. 2026 Jun;36(6):e70312.

18.	Fell JM, Hearris MA, Ellis DG, Moran JEP, Jevons EFP, Owens DJ, et al. Carbohydrate improves exercise capacity but does not affect subcellular lipid droplet morphology, AMPK and p53 signalling in human skeletal muscle. J Physiol. 2021 Jun;599(11):2823-49.

19.	Bradley WJ, Morehen JC, Haigh J, Clarke J, Donovan TF, Twist C, et al. Muscle glycogen utilisation during Rugby match play: Effects of pre-game carbohydrate. J Sci Med Sport. 2016 Dec;19(12):1033-8.

20.	Jones LA, Leung L, Barrett JS, Fortis HO, Strauss JA, Shepherd SO. New Zealand blackcurrant extract augments muscle glycogen storage at rest and enhances intramuscular triglyceride degradation during prolonged exercise. Eur J Appl Physiol. 2026 Mar;126(3):1857-72.

21.	Jansson E, Kaijser L. Effect of diet on muscle glycogen and blood glucose utilization during a short-term exercise in man. Acta Physiol Scand. 1982 Jul;115(3):341-7.

22.	Adamo KB, Tarnopolsky MA, Graham TE. Dietary carbohydrate and postexercise synthesis of proglycogen and macroglycogen in human skeletal muscle. Am J Physiol. 1998 Aug;275(2):E229-34.

23.	Bradley WJ, Hannon MP, Benford V, Morehen JC, Twist C, Shepherd S, et al. Metabolic demands and replenishment of muscle glycogen after a rugby league match simulation protocol. J Sci Med Sport. 2017 Sep;20(9):878-83.

24.	Hickner RC, Fisher JS, Hansen PA, Racette SB, Mier CM, Turner MJ, et al. Muscle glycogen accumulation after endurance exercise in trained and untrained individuals. J Appl Physiol (1985). 1997 Sep;83(3):897-903.

25.	Roberts PA, Fox J, Peirce N, Jones SW, Casey A, Greenhaff PL. Creatine ingestion augments dietary carbohydrate mediated muscle glycogen supercompensation during the initial 24 h of recovery following prolonged exhaustive exercise in humans. Amino Acids. 2016 Aug;48(8):1831-42.

26.	Tomcik KA, Camera DM, Bone JL, Ross ML, Jeacocke NA, Tachtsis B, et al. Effects of Creatine and Carbohydrate Loading on Cycling Time Trial Performance. Med Sci Sports Exerc. 2018 Jan;50(1):141-50.

27.	Maunder E, Bradley HE, Deane CS, Hodgson AB, Jones M, Joanisse S, et al. Effects of short-term graded dietary carbohydrate intake on intramuscular and whole body metabolism during moderate-intensity exercise. J Appl Physiol (1985). 2021 Jul 1;131(1):376-87.

28.	Elbourne DR, Altman DG, Higgins JP, Curtin F, Worthington HV, Vail A. Meta-analyses involving cross-over trials: methodological issues. Int J Epidemiol. 2002 Feb;31(1):140-9.

29.	Rauch LH, Rodger I, Wilson GR, Belonje JD, Dennis SC, Noakes TD, et al. The effects of carbohydrate loading on muscle glycogen content and cycling performance. Int J Sport Nutr. 1995 Mar;5(1):25-36.

30.	Walker JL, Heigenhauser GJ, Hultman E, Spriet LL. Dietary carbohydrate, muscle glycogen content, and endurance performance in well-trained women. J Appl Physiol (1985). 2000 Jun;88(6):2151-8.

31.	Kavouras SA, Troup JP, Berning JR. The influence of low versus high carbohydrate diet on a 45-min strenuous cycling exercise. Int J Sport Nutr Exerc Metab. 2004 Feb;14(1):62-72.

32.	Schytz CT, Ortenblad N, Birkholm TA, Plomgaard P, Nybo L, Kolnes KJ, et al. Lowered muscle glycogen reduces body mass with no effect on short-term exercise performance in men. Scand J Med Sci Sports. 2023 Jul;33(7):1054-71.

33.	Arkinstall MJ, Bruce CR, Clark SA, Rickards CA, Burke LM, Hawley JA. Regulation of fuel metabolism by preexercise muscle glycogen content and exercise intensity. J Appl Physiol (1985). 2004 Dec;97(6):2275-83.

34.	Burke LM, Hawley JA, Schabort EJ, St Clair Gibson A, Mujika I, Noakes TD. Carbohydrate loading failed to improve 100-km cycling performance in a placebo-controlled trial. J Appl Physiol (1985). 2000 Apr;88(4):1284-90.

35.	Hawley JA, Palmer GS, Noakes TD. Effects of 3 days of carbohydrate supplementation on muscle glycogen content and utilisation during a 1-h cycling performance. Eur J Appl Physiol Occup Physiol. 1997;75(5):407-12.

36.	Higgins JPT TJ, Chandler J, Cumpston M, Li T, Page MJ, Welch VA (editors). . Cochrane Handbook for Systematic Reviews of Interventions version 6.5 (updated August 2024). Available from <www.training.cochrane.org/handbook>: Cochrane; 2024.

37.	Tarnopolsky MA, Atkinson SA, Phillips SM, MacDougall JD. Carbohydrate loading and metabolism during exercise in men and women. J Appl Physiol (1985). 1995 Apr;78(4):1360-8.

38.	Tarnopolsky MA, Zawada C, Richmond LB, Carter S, Shearer J, Graham T, et al. Gender differences in carbohydrate loading are related to energy intake. J Appl Physiol (1985). 2001 Jul;91(1):225-30.

39.	Widrick JJ, Costill DL, Fink WJ, Hickey MS, McConell GK, Tanaka H. Carbohydrate feedings and exercise performance: effect of initial muscle glycogen concentration. J Appl Physiol (1985). 1993 Jun;74(6):2998-3005.
